# CoRAL accurately resolves extrachromosomal DNA genome structures with long-read sequencing

**DOI:** 10.1101/gr.279131.124

**Published:** 2024-09

**Authors:** Kaiyuan Zhu, Matthew G. Jones, Jens Luebeck, Xinxin Bu, Hyerim Yi, King L. Hung, Ivy Tsz-Lo Wong, Shu Zhang, Paul S. Mischel, Howard Y. Chang, Vineet Bafna

**Affiliations:** 1 Department of Computer Science and Engineering, University of California San Diego, La Jolla, California 92093, USA;; 2 Center for Personal Dynamic Regulomes, Stanford University, Stanford, California 94305, USA;; 3 Bioinformatics Undergraduate Program, School of Biological Sciences, University of California San Diego, La Jolla, California 92093, USA;; 4 Howard Hughes Medical Institute, Stanford University, Stanford, California 94305, USA;; 5 Department of Pathology, Stanford University School of Medicine, Stanford, California 94305, USA;; 6 Sarafan Chemistry, Engineering, and Medicine for Human Health (Sarafan ChEM-H), Stanford University, Stanford, California 94305, USA;; 7 Department of Dermatology, Stanford University School of Medicine, Stanford, California 94305, USA;; 8 Department of Genetics, Stanford University, Stanford, California 94305, USA;; 9 Halıcıoğlu Data Science Institute, University of California San Diego, La Jolla, California 92093, USA

## Abstract

Extrachromosomal DNA (ecDNA) is a central mechanism for focal oncogene amplification in cancer, occurring in ∼15% of early-stage cancers and ∼30% of late-stage cancers. ecDNAs drive tumor formation, evolution, and drug resistance by dynamically modulating oncogene copy number and rewiring gene-regulatory networks. Elucidating the genomic architecture of ecDNA amplifications is critical for understanding tumor pathology and developing more effective therapies. Paired-end short-read (Illumina) sequencing and mapping have been utilized to represent ecDNA amplifications using a breakpoint graph, in which the inferred architecture of ecDNA is encoded as a cycle in the graph. Traversals of breakpoint graphs have been used to successfully predict ecDNA presence in cancer samples. However, short-read technologies are intrinsically limited in the identification of breakpoints, phasing together complex rearrangements and internal duplications, and deconvolution of cell-to-cell heterogeneity of ecDNA structures. Long-read technologies, such as from Oxford Nanopore Technologies, have the potential to improve inference as the longer reads are better at mapping structural variants and are more likely to span rearranged or duplicated regions. Here, we propose Complete Reconstruction of Amplifications with Long reads (CoRAL) for reconstructing ecDNA architectures using long-read data. CoRAL reconstructs likely cyclic architectures using quadratic programming that simultaneously optimizes parsimony of reconstruction, explained copy number, and consistency of long-read mapping. CoRAL substantially improves reconstructions in extensive simulations and 10 data sets from previously characterized cell lines compared with previous short- and long-read-based tools. As long-read usage becomes widespread, we anticipate that CoRAL will be a valuable tool for profiling the landscape and evolution of focal amplifications in tumors.

Oncogene amplification is one of the most common events in tumorigenesis, contributing to tumor initiation and progression (Beroukhim et al. 2010; Steele et al. 2022). Often, these amplifications are mediated by the formation of circular, megabase-scale extrachromosomal DNA (ecDNA) (Turner et al. 2017; Wu et al. 2019; Kim et al. 2020). Previous studies have underscored the importance of ecDNA in driving tumor formation (Luebeck et al. 2023), evolution (Lange et al. 2022), oncogene-mediated gene regulation (Hung et al. 2021; Zhu et al. 2021), and drug resistance (Nathanson et al. 2014; Lange et al. 2022). Thus, profiling the genetic and structural landscape of small, focal amplifications (typically < 10 Mb), such as ecDNA, in tumors is critical for understanding the mechanisms of tumor progression and developing more effective therapies.

Because of the large and complex genomes of ecDNA, it remains challenging to accurately infer the set of “amplicon” structures present in tumors (Deshpande et al. 2019; Luebeck et al. 2020; Chapman et al. 2023). Existing approaches rely on paired-end, short-read (Illumina) sequencing to identify amplicons from copy-number profiles and breakpoints, which then can be represented with an edge-weighted *breakpoint graph*; ecDNAs can subsequently be extracted as cycles from the breakpoint graph (Bafna and Pevzner 1996; Alekseyev and Pevzner 2009; Lin et al. 2014; Deshpande et al. 2019; Hadi et al. 2020). Despite the success of these approaches in predicting ecDNA presence in cancer samples (Deshpande et al. 2019; Kim et al. 2020; Luebeck et al. 2023), short-read reconstructions have several limitations. First, short-read approaches struggle to handle the highly rearranged nature of ecDNA and accurately detect breakpoints, especially in repetitive or low-complexity regions. Second, because ecDNA can contain multiple copies of large segments that are unique in the reference (e.g., Fig. 1A), short-read data are limited in their ability to phase distant breakpoints correctly. Therefore, multiple collections of paths or cycles in the breakpoint graph can explain the increased copy number equally well, masking the true structure (Fig. 1C). Third, heterogeneity of ecDNA structures might result in multiple overlapping focal amplifications derived from the same genomic regions. To address these shortcomings of short-read technology, existing methods (e.g., AmpliconArchitect [AA]) (Deshpande et al. 2019; Hung et al. 2022) must use heuristics: for example, extracting cycles with the highest copy number iteratively from a breakpoint graph, until a large fraction of the aggregate copy number is explained. Although these heuristic strategies return multiple small cycles (Fig. 1C) that can later be recombined (Hung et al. 2022), they are still constrained by the intrinsic limitations of short-read technologies to identify structural variation and phase together distant breakpoints.

**Figure 1.**
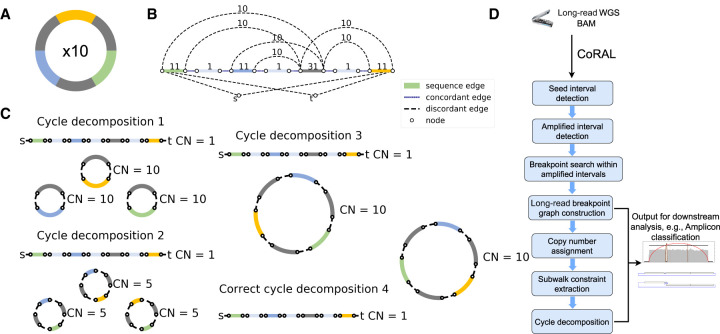
Long-read-based ecDNA reconstruction. (*A*) Native ecDNA structure and copy number. (*B*) Cartoon of the breakpoint graph derived from the ecDNA in *A*. Sequence edges represent segments of the reference genome. Concordant edges connect consecutive sequences with respect to the reference genome order, and discordant edges connect nonconsecutive genome segments. Nodes are created at the endpoints of each sequence edge and include source and sink nodes, *s* and *t*. (*C*) Multiple collections of decomposed paths and cycles from the breakpoint graph explain the changes in copy number and observed SVs. Long reads that span regions of high multiplicity can help resolve the correct cycle. (*D*) Overview of the CoRAL method.

Long reads have the potential to resolve these challenges. Recent research efforts utilized Oxford Nanopore reads to reconstruct simple ecDNA, building on off-the-shelf de novo assembly (Helmsauer et al. 2020). However, de novo assembly methods often make choices based on underlying assumptions that do not hold: For example, they assume a diploid genome and that regions of high multiplicity are small enough to be spanned by long reads. However, the heterogeneity of ecDNA structures violates the assumption of ploidy, and the long segments of high multiplicity (typically 10 kb–1 Mb) in ecDNA are infrequently spanned by a single read, unlike the repetitive regions encountered in genome assembly, such as long interspersed nucleotide elements (LINEs), which are in the 10 kb range. Concurrent with our method proposed below, a new approach, Decoil (Giurgiu et al. 2024), also aims at reconstructing ecDNA structures with long reads. However, it does not separate multiple distinct focal amplifications in one tumor sample and uses a similar “simple cycle extraction and combining” heuristic designed for short reads to reconstruct ecDNAs with high multiplicity segments. An alternative methodology utilizes optical mapping (OM) (Cao et al. 2014) to sequence large (>200 kbp) DNA fragments that span a limited number of the high multiplicity regions (Luebeck et al. 2020). Although good for scaffolding, these data cannot precisely detect breakpoints, identify small structural variations, or resolve nontemplated sequence, and they work best in conjunction with short-read methods.

Here, we propose Complete Reconstruction of Amplifications with Long reads (CoRAL), an algorithm for reconstructing ecDNA amplicon sequence and structure from long reads (such as those from Oxford Nanopore Technologies or Pacific Biosciences [PacBio]). CoRAL builds a distinct breakpoint graph for each focally amplified region, as well as extracts cycles (and walks) from the breakpoint graph representing ecDNA (and the potential focally amplified genomes). In cases in which the reads are not always long enough to span the high multiplicity regions, CoRAL reconstructs likely cyclic architectures using quadratic programming that simultaneously optimizes parsimony of reconstruction, explained copy number, and consistency of long-read mapping. Through extensive benchmarks on simulated data and previously characterized cell lines, we report that CoRAL substantially improves breakpoint detection and inference of the order of complex segments on ecDNA over long-read-based Decoil (Giurgiu et al. 2024) and short-read-based AA (Deshpande et al. 2019) methods.

## Results

### An overview of the CoRAL method

For better exposition of the results, we first provide a brief description of the method. A pictorial overview can be found in Figure 1D, and details can be found in the Methods and Supplemental Methods S2–S4. CoRAL takes mapped long reads (in BAM format) as input and begins by identifying focally amplified *seed intervals*. The seed intervals can be provided directly or can be derived from whole-genome CNV calls (e.g., with third-party tools like CNVkit) (Talevich et al. 2016) of mapped long reads. From the CNV calls, CoRAL selects genomic segments with minimum thresholds on copy number and aggregate size as seed intervals (Supplemental Methods S2).

CoRAL uses these seed intervals to construct a copy-number-weighted breakpoint graph separately for each amplified region. The graph construction starts with exploring all *amplified intervals* connected to the seed intervals through discordant edges given by chimeric long-read mappings. Once all amplified intervals are identified for each focal amplification, a graph structure is organized by CoRAL to include the genome segments (sequence edges) from the amplified intervals, the concordant edges that join neighboring genome segments, and also the discordant edges within the amplified intervals and those connecting different amplified intervals. Once the graph structure is fixed, CoRAL recomputes the *copy number* for each edge, which can best explain the long-read coverage on each edge, while maintaining a balance of copy number between sequence edges and concordant/discordant edges incident on nodes (see Methods) (Supplemental Methods S3).

As its key step, CoRAL reconstructs potential ecDNA structures in the breakpoint graph by extracting a minimum number of *cycles and walks* from the graph, allowing duplication of nodes (e.g., Fig. 1C), in which cycles represent the potential ecDNA species, and walks represent linearly amplified or rearranged genomic segments. Each cycle/walk is associated with a positive weight—corresponding to the copy number—so that the sum of the length-weighted edges of extracted walks explains a large fraction of the total copy number of the edges in the breakpoint graph. In addition, CoRAL takes advantage of the fact that long reads may span several breakpoints and incorporates these reads as *subwalk constraints*. In its cycle extraction, CoRAL also requires a majority of the subwalk constraints to be satisfied by the resulting cycles and walks, thus leveraging the power of long reads. CoRAL uses quadratically constrained programming to solve a multiobjective optimization that minimizes the number of cycles/walks while maximizing the explained length-weighted copy number and the number of subwalk constraints (Methods) (Supplemental Methods S4). It finally outputs the reconstructed breakpoint graphs for each focal amplification in the sample, as well as the associated cycles/walks from the graph. It also optionally outputs stylistic visualizations of the breakpoint graphs and cycles, as shown in subsequent results.

### Simulation benchmarks

We first assessed the effectiveness of amplicon reconstruction algorithms using simulated sequencing data from synthetic amplicon structures (Supplemental Methods S5; Supplemental Tables S1, S2). To capture the diversity of ecDNA amplicons observed in patient tumors and cell lines, we simulated 75 distinct cyclic structures with varying numbers of breakpoints (between one and 20) from one of three origins: *episomal*, in which a contiguous region of the genome is excised from a chromosome; *chromothripsis*, in which a mitotic defect leads to the shattering of a lagging chromosome and ecDNA formation (Ly et al. 2017; Shoshani et al. 2021); or, finally, *2-foldback*, in which extruding double-stranded DNA from a stalled replication fork is broken off as ecDNA (Passananti et al. 1987). Our simulated ecDNAs additionally included internal structural variants in the form of insertions, deletions, duplications, and inversions (for more detailed description of the simulation process, see Supplemental Methods S5; for the data, see Supplemental Table S1). Subsequently, each test data set was generated by randomly selecting between one and five amplicon structures (from the pool of 75 synthetic amplicons). Reads from long-read (using Nanosim) (Yang et al. 2017) and Illumina short-read, paired-end technologies (using Mason) (Holtgrewe 2010) were simulated from these amplicons at one of three coverages (50×, 100×, or 250× coverage; or approximate copy numbers of 7, 15, or 37, respectively) and merged with reads from one of five simulated normal, diploid genomes (each with ∼13× coverage). A total of 50 test data sets were simulated in this fashion and used for benchmarking amplicon reconstruction (Supplemental Table S2).

**Figure 2.**
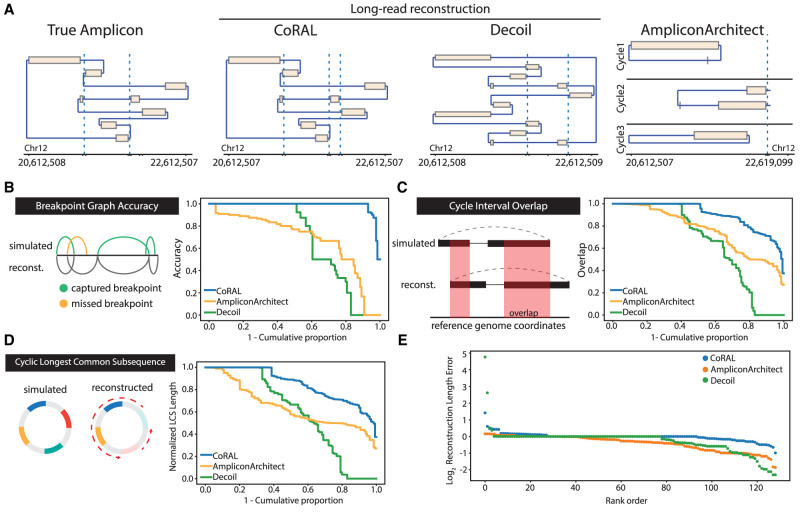
Overview of simulation benchmarking. (*A*) True structure compared with CoRAL, Decoil, and AA reconstructions for an example amplicon (Episomal, eight observed breakpoints). (*B*–*E*) Cumulative distributions of CoRAL, Decoil, and AA reconstructions across all simulations for breakpoint graph accuracy (*B*), cycle interval overlap (*C*), cyclic longest common subsequence (*D*), and rank-order distribution of reconstruction length error (*E*). Empirical cumulative densities are reported for *B*, *C*, and *D*; and each point in *E* corresponds to a simulated amplicon. For more detailed information, see Supplemental Methods S7.

From these inputs, ecDNA was reconstructed using simulated long reads provided to CoRAL and Decoil (Giurgiu et al. 2024)—a separate long-read amplicon reconstruction tool—or simulated short reads provided to AA. In most cases, the *heaviest* CoRAL cycle, defined as the cycle with the largest length-weighted copy number, was better at recapitulating the true architecture compared with the AA cycle (e.g., Fig. 2A). We systematically evaluated the accuracy of the best reconstruction *W*_*r*_ (as defined as the highest-scoring reconstruction with respect to a particular statistic) against a true cycle *W*_*t*_ using four additional measures defined briefly below (Fig. 2B–E; for more detailed definitions, see Supplemental Methods S7):

1. **Breakpoint graph accuracy** reports the proportion of discordant edges that agree, up to a tolerance of 100 bp, between the true breakpoint graph *G*_*t*_ and reconstructed breakpoint graph *G*_*r*_.
2. **Cycle interval overlap** measures the Jaccard index, weighted by the number of nucleotides, of the genomic intervals defined by *W*_*t*_ and *W*_*r*_.
3. **Cyclic longest common subsequence (LCS)** measures the length of the longest common subsequence contained in *W*_*t*_ and *W*_*r*_ after eliminating intervals that are not found in both, normalized to the length of *W*_*t*_.
4. **Reconstruction length error** reports the difference in amplicon lengths between *W*_*r*_ and *W*_*t*_, normalized by the true amplicon length *W*_*t*_. We report *log*_2_-scaled values.

Across the 50 simulated data sets, we observed consistently improved reconstruction of CoRAL over AA and Decoil for all four measures (Fig. 2B–E). Notably, 93% of CoRAL reconstructions perfectly recapitulated the ground-truth breakpoint graph compared with 51% for Decoil and 4% for AA (Fig. 2B). These results underscore the improved mapping of structural variants with long reads.

Although CoRAL outperformed Decoil and AA in all four measures, both AA and Decoil capture many critical aspects of the amplicon, such as including the most amplified intervals (Fig. 2C) and capturing the true ordering of the segments (Fig. 2D). Mostly, CoRAL's improved performance is reflected in reconstructed cycle lengths that are most similar to the true cycle (Fig. 2E). In addition, we observe that both AA and CoRAL tend to produce a main cycle that accounts for a large fraction of length-weighted copy number (Supplemental Fig. S1) and that this weight ratio is correlated with cycle reconstruction accuracy (Supplemental Fig. S2). Through these analyses, we also noted several examples in which the interval ordering is incorrect despite near-perfect recovery of breakpoint graph and interval overlap (Supplemental Fig. S3), reflecting the technological limitations of reads that were not long enough to resolve the true order of segments.

We also compared reconstruction performance as a function of the complexity of amplicons (number of segments, or sequence edges), sequence coverage, their formation context, and level of duplication (or multiplicity). We observed that the number of segments in the true amplicon had modest effects on reconstruction accuracy (Supplemental Fig. S4), as did coverage (and by extension copy number) (Supplemental Fig. S5). These observations suggest that all algorithms, but especially CoRAL, can accurately reconstruct complex ecDNAs at low copy numbers (e.g., less than seven). We additionally observed that increasing levels of segmental or breakpoint multiplicity often resulted in poorer reconstruction accuracy for all methods tested, although CoRAL remained mostly robust (Supplemental Fig. S6). However, in considering the various contexts in which ecDNA can form (Bafna and Mischel 2022), we observed substantial performance differences: Generally, we observed that chromothripsis amplicons were most difficult for AA and CoRAL, with Decoil modestly outperforming CoRAL; conversely, 2-foldback amplicons were most difficult for Decoil. These observations highlight the importance of accurately detecting structural variants, which is greatly enhanced with long reads but can be nevertheless challenging depending on the complexity of breakpoints (Supplemental Fig. S7).

### Amplicon reconstruction in cell lines

Next, we evaluated amplicon reconstruction using matched Nanopore long-read sequencing and Illumina paired-end short-read sequencing in seven previously characterized cell lines spanning a range of cancer types and amplifications (for a summary, see Table 1; Supplemental Table S3): COLO320(-DM, -HSR), PC3(-DM, -HSR), GBM39(-HSR), and CHP-212. Of these seven cell lines, there are three isogenic pairs in which the amplified oncogene is located on chromosomal homogeneously staining regions (HSRs) while maintaining the core cyclic structure, as opposed to ecDNA (e.g., COLO320-HSR vs. COLO320-DM). Additionally, we assessed reconstruction in four recently monoclonalized versions of these cell lines (PC3-DM, PC3-HSR, GBM39ec, and COLO320-DM). Together, this resulted in a matched Nanopore and Illumina data for 10 samples for analysis.

**Table 1.** Overview of cell lines profiled in this study

Cell line name	Cancer type	Gene(s)	Amplification type	Monoclonal status	Source
PC3DM (mono)	Prostate	*MYC*	ecDNA	Yes	This study
PC3HSR (mono)	Prostate	*MYC*	HSR	Yes	This study
GBM39 (mono)	Glioblastoma	*EGFRvIII*	ecDNA	Yes	This study
COLO320-DM (mono)	Colorectal	*MYC*	ecDNA	Yes	This study
COLO320-DM	Colorectal	*MYC*	ecDNA	No	(Hung et al. 2021)
COLO320-DM	Colorectal	*MYC*	ecDNA	No	(Wu et al. 2019)
GBM39	Glioblastoma	*EGFRvIII*	ecDNA	No	(Wu et al. 2019)
CHP-212	Neuroblastoma	*MYCN, TRIB2*	ecDNA	No	(Helmsauer et al. 2020)
COLO320-HSR	Colorectal	*MYC*	HSR	No	(Wu et al. 2019)
GBM39-HSR	Glioblastoma	*EGFRvIII*	HSR	No	(Wu et al. 2019)

Cell type name, cancer type, subset of important amplified oncogenes, amplification type, monoclonal status, and source. (HSR) Homogeneously staining region, (ecDNA) extrachromosomal DNA.

### CoRAL accurately predicts the existence of ecDNA

We ran the AmpliconClassifier (Luebeck et al. 2023) method to reconfirm the cyclic structure of the ecDNA amplicons in all samples. AmpliconClassifier parses the breakpoint graph and identifies subgraphs as being cyclic (or ecDNA), breakage fusion bridge, heavily rearranged, or linear-rearranged (Kim et al. 2020). CoRAL identified altogether 60 amplicons in the 10 cell lines, including the main ecDNA (or HSR) amplicon in each sample. AmpliconClassifier consistently classified the main ecDNA amplicon as cyclic with the breakpoint graphs constructed by CoRAL using long reads and AA using short reads, indicating the existence of ecDNA (or HSR). Long-read sequencing did not identify new ecDNA amplicons, and it did not fail to detect previously confirmed ecDNA amplicons in the cell line samples.

### CoRAL cycles better explain the copy numbers in ecDNA amplicons

**Figure 3.**
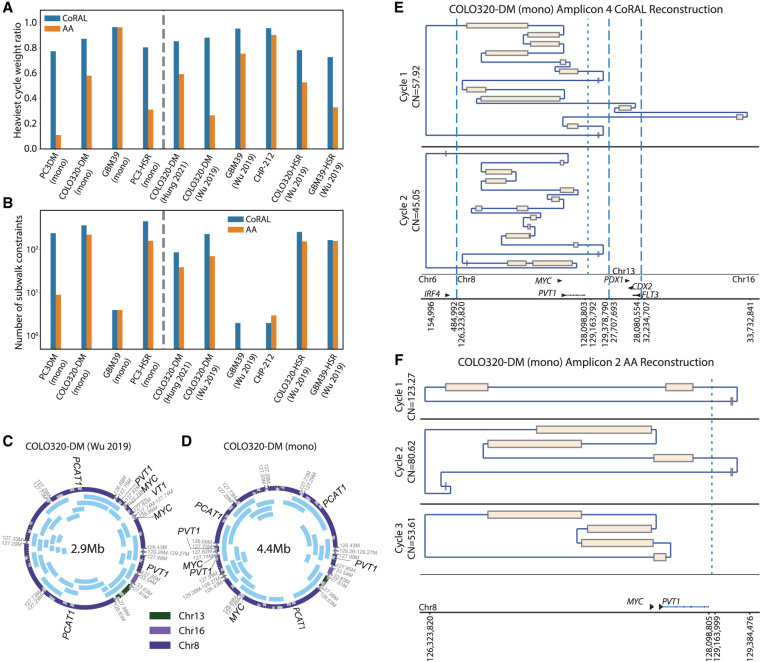
Amplicon reconstruction in cell lines. (*A*) Fraction of length-weighted copy numbers given by the three heaviest cycles, reported by CoRAL and AA. (*B*) Number of satisfied subwalk constraints by CoRAL and AA, in cell lines. (*C*) The cycle with largest length-weighted CN from previously published COLO320-DM. (*D*) The cycle with largest length-weighted CN from monoclonalized COLO320-DM. In *C* and *D*, each blue arc within the cycle indicates a subwalk constraint satisfied by that cycle. (*E*) The two cycles with largest length-weighted copy numbers by CoRAL. (*F*) The three cycles with largest length-weighted copy numbers by AA.

To benchmark the reconstruction quality of CoRAL and AA in cell lines, we computed the fraction of length-weighted copy numbers in the breakpoint graph given by the *k*-heaviest cycles, which we previously observed correlated with accuracy (with *k* = 1), for *k* = 3 and *k* = 1, in each of the 10 ecDNA cell lines (Fig. 3A; Supplemental Fig. S8A; for details of the statistic, see Supplemental Methods S7). Consistent with simulated data, these results demonstrated that CoRAL explains a higher fraction of the length-weighted copy number with fewer cycles. Across all samples, the copy number explained by the three heaviest cycles was substantially higher for CoRAL compared with AA (Fig. 3A). The reconstructed cycles of COLO320-DM (Wu et al. 2019), the monoclonal COLO320-DM (mono), and the shallow coverage COLO320-DM (Hung et al. 2021) showed consistent heaviest cycle weight ratio (Fig. 3A; Supplemental Fig. S8A), shared many structural features, and contained a similar subset of genes (Fig. 3C,D; Supplemental Table S4), even as they showed some differences in the reconstructed amplicons (Supplemental Fig. S9). These differences could reflect differences in intrinsic heterogeneity or evolution of the cell line over time, which also resulted in a lower heaviest-cycle-weight ratio in COLO320-DM cells and its isogeneic pair COLO320-HSR, in comparison to the GBM39 and CHP-212 cell lines with a single dominating ecDNA structure (Supplemental Fig. S8A; Wu et al. 2019; Helmsauer et al. 2020).

### CoRAL cycles satisfy more subwalk constraints

In its optimization, CoRAL takes advantage of the fact that long reads may span several breakpoints and incorporates these reads as *subwalk constraints* that can be satisfied during cycle decomposition and lend support for accurate reconstruction. As such, we mapped each long-read subwalk constraint to each AA cycle and checked if the subwalk constraint can also be satisfied by that cycle. CoRAL satisfied more subwalk constraints compared with AA (Fig. 3B; Supplemental Fig. S8B), especially for the complex amplicons. For example, CoRAL satisfied 1.5× and 25× more subwalk constraints in COLO320-DM (mono) and PC3-DM (mono), respectively. Together, these subwalk constraints support most junctions of the amplicon (e.g., see Fig. 3C,D), thereby taking advantage of the long reads that span multiple breakpoints. Nevertheless, no reconstruction satisfied all subwalk constraints in either CoRAL or AA, consistent with the high heterogeneity of ecDNA structure in samples.

### CoRAL cycles enable the study of critical aspects of amplicon structures

Reconstruction supported by long-read subwalk constraints additionally enabled the study of critical aspects of the amplicon structures. As one example, Figure 3, E and F, shows the reconstruction of the two heaviest CoRAL and the three heaviest AA cycles, respectively, for monoclonal COLO320-DM. To note, the monoclonal COLO320-DM is a recently derived line from a parental line in which previous experiments integrating WGS, OM, and in vitro ecDNA digestion revealed an ecDNA structure of ∼4.3 Mb (Hung et al. 2021). Here, the automated reconstruction of monoclonal COLO320-DM using CoRAL also revealed an ecDNA of size 4.4 Mbp (Fig. 3D), which shared many structural features with the previous reconstruction.

One distinct feature of the COLO320-DM *MYC* amplicon is the overexpression of a fusion transcript consisting of a truncated, 5′ portion of the lncRNA *PVT1* fused to the second exon of the *MYC* oncogene (Hung et al. 2021). This is despite *PVT1* being positioned downstream from *MYC* in the reference genome. As expected, CoRAL reconstruction of COLO320-DM includes a breakpoint that connects a truncated, 5′ portion of *PVT1* upstream of exon 2 of *MYC*, thereby explaining the fused transcript. Notably, both CoRAL and AA detected the *PVT1*-*MYC* fusion breakpoint in all COLO320-DM samples; however, CoRAL's cycle decomposition included this breakpoint in the heaviest (largest length-weighted CN) cycle across multiple COLO320-DM samples (Fig. 3C–E; Supplemental Table S4). AA did not include the breakpoint in the three heaviest cycles (Fig. 3F); instead, it reports a smaller cycle of a size of ∼90 kbp containing the breakpoint by itself. Furthermore, subwalk constraints owing to long reads linked truncated *PVT1* and *MYC* on a single molecule. Correspondingly, CoRAL reconstructions of cycles in COLO320-DM (mono), COLO320-DM (Hung et al. 2021), and COLO320-DM (Wu et al. 2019) all showed the three elements in a single cycle (Fig. 3C,D; Supplemental Table S4).

We additionally observed that subwalk constraints and CoRAL's cycle reconstructions support a coamplification of the ncRNA *PCAT1* and *MYC* on COLO320-DM ecDNA (Fig. 3E,F). Previous DNA FISH experiments also confirmed the coexistence of these genes on COLO320-DM (extended Fig. 4G) (Hung et al. 2021). The *PCAT1* ncRNA is known to repress *BRCA2* (Prensner et al. 2014b), activate *MYC* (Prensner et al. 2014a), promote cell proliferation (Xiong et al. 2019), and is upregulated in prostate, colorectal, and other cancers (Xiong et al. 2019). Thus, these CoRAL cycle reconstructions are also consistent with the regulatory and pro-oncogenic roles of *MYC* and *PCAT1*. Together, these results highlight the advantages of CoRAL in reconstructing complex ecDNAs in cell lines and may enable new biological insights into the coamplification of genetic elements on the same ecDNA molecule.

### CoRAL requires comparable computational resources to AA

We finally compared the computational resources required by CoRAL and AA to reconstruct all amplicons in these cell lines. To perform a fair test, we ran CoRAL and AA on the same Ubuntu system (2× Intel Xeon X5680 CPU, and 128 GB RAM). Importantly, we observed that the total running time and memory of CoRAL was comparable to that of AA for reconstructing the amplicons, even if an MIQCP was solved for each amplicon (Supplemental Fig. S10). The most complex sample, COLO320-HSR (Wu et al. 2019), was completed in <22 h (8 × 10^5^ sec) for CoRAL. Furthermore, we found that most focal amplifications except ecDNA are relatively easy to resolve, with the resulting breakpoint graphs being small: Out of the 60 amplicons detected by CoRAL across all samples, only eight required greedy MIQCP, including seven of the 10 total ecDNA amplicons.

## Discussion

Our results suggest that long-read guided reconstruction greatly improves ecDNA structure resolution, both in individual detection of breakpoints and in the accuracy of the large-scale predicted structure. The constrained optimization performed by CoRAL reconstructs plausible structures based on selecting a minimum number of cycles that are consistent with the constraints provided by long reads, and together, the cycles explain most of the copy number of the amplicon. On simulated data, most structures were correctly predicted, and even when they were not, they were only slightly rearranged from the true structure. Similarly, in experimental data from cancer cell lines, the three heaviest reconstructed structures typically explained most of the copy number. In most cases, the reconstruction provides a reasonable template for downstream functional studies, including analysis of regulatory rewiring and chromatin conformation. Of note, CoRAL's approach can be seamlessly employed for any long-read sequencing technology, such as Oxford Nanopore Technologies or PacBio, in which longer reads will always improve breakpoint detection and amplicon reconstruction.

It is important to note, however, that long reads by themselves are not a panacea, and amplicon reconstruction is different than genome assembly. In diploid genomes, only two haploid structures are possible, and the repeated regions are easily spanned by current long-read technology, except in a few highly repetitive regions. In contrast, larger regions can occur with multiple copies on a single ecDNA, making it hard to resolve into one correct structure. Moreover, heterogeneity of ecDNA may lead to many structures being present. The ecDNA structures resolved by CoRAL may only reflect the most abundant structures. Moreover, because of the minimization of cycle counts, it is possible that the heaviest cycle given by CoRAL glues together smaller ecDNA cycles that share the same segments. To avoid such cases, we limit the times that each discordant edge can be traversed in a cycle or walk based on empirical observations. Reconstructions from simulated and real ecDNA amplicons (e.g., COLO320DM) suggested similar cycle sizes to either ground-truth or previous characterizations. These considerations will be revised as additional data become available.

Thus, we highlight a few avenues for extending and improving CoRAL. First, when a sample has concurrent short-read sequencing data, one may explore if incorporating low-coverage long reads (<5×) is sufficient for a hybrid reconstruction. However, because of the rapid evolution of cancer genomes and spatial heterogeneity of tumor samples, the benefit of such an approach may only exist when short and long reads are simultaneously generated from the same biospecimen. Second, CoRAL can be extended to identify the architectures of chromosomal amplicons, such as breakage fusion bridge cycles, and ecDNA that have reintegrated into the genome. Because the reconstruction methods use only abstractions relating to path constraints and explained copy number, they can be adapted to other amplifications readily, and this will also be a focus of future studies. Third, as our understanding of amplicon structure grows with experimentally verified structures, that information can be used to improve the constraint space and optimization criteria for CoRAL and to enhance the simulations of ecDNA or other chromosomal amplifications.

Previous state-of-the-art tools using short reads like AA (Deshpande et al. 2019) are very accurate in determining if a focal amplification is mediated by ecDNA formation and in determining the amplified regions. However, they have difficulties in reconstructing the full structure or in determining all the regions that participate in one ecDNA molecule. These challenges are partially resolved by targeted deep profiling of a specific subset of amplicons at the expense of not observing the full amplification landscape (Hung et al. 2022). CoRAL not only offers improvements as a standalone tool but can also be used in conjunction with the targeted approaches, either by refining existing reconstructions or by providing more accurate and unambiguous reconstructions of complex amplicons in targeted enrichment protocols. In summary, CoRAL will be a valuable tool in the arsenal for analyzing complex focal amplifications, such as ecDNA, in tumor genomes, especially as long-read technologies continue to offer cheaper, longer, and more accurate reads.

## Methods

CoRAL takes mapped long reads (in BAM format) as input, constructs a copy-number-weighted breakpoint graph, decomposes the breakpoint graph into a collection of cycles or paths, and outputs the reconstructed breakpoint graph as well as the resulting cycles/paths from decomposition of the breakpoint graph. A pictorial overview of CoRAL procedure is given in Figure 1D.

Below, we start with an abstract definition of the breakpoint graph followed by a high-level description of the construction. The copy-number-weighted breakpoint graph (Bafna and Pevzner 1996; Alekseyev and Pevzner 2009; Lin et al. 2014; Deshpande et al. 2019; Hadi et al. 2020), denoted by $G=(V,E=E_{s}\cup E_{c}\cup E_{d},\mathrm{CN})$, encodes a collection of nonoverlapping intervals on a given reference genome, which are amplified, reordered, or reoriented. A brief description is provided here, with details in Supplemental Methods S1:

- Each *v* ∈ *V* represents the start or end coordinate of an interval, or the special *source* nodes *s*, *t* (defined below). Let *l*_*v*_ denote the location of node *v*.
- *E*_*s*_ represents *sequence edges*, which join the start and end coordinates of an interval.
- *E*_*c*_ represents *concordant edges* so that (*u*, *v*) ∈ *E*_*c*_ if *l*_*v*_ − *l*_*u*_ = 1, where *v* is the start coordinate of the canonically larger interval on the reference genome represented by a sequence edge.
- *E*_*d*_ represents *discordant edges*, generated when (sufficient) reads map to discordant intervals. Thus, (*u*, *v*) ∈ *E*_*d*_ if |*l*_*v*_ − *l*_*u*_| ≠ 1 if the read connecting *u* to *v* changes orientation or if the nodes are on different chromosomes. A discordant edge could connect the start (or end) coordinate of an interval to itself (an inverted duplication or *foldback*).
- All edges are weighted using the real-valued function CN: *E* → ℚ^+^ denoting the *copy number*. The CN is computed based on an assumption of diploidy for the majority of base pairs on the genome. We require that the CN assignment be “balanced,” for each (*u*, *v*) ∈ *E*_*s*_, as follows:
  (1) $$\underset{(w,u)\in E_{c}\cup E_{d}}{\sum }\mathrm{CN}(w,u)=\mathrm{CN}(u,v)=\underset{(v,w)\in E_{c}\cup E_{d}}{\sum }\mathrm{CN}(v,w),\;\forall (u,v)\in E_{s}.$$

By definition, each node in a breakpoint graph is connected to a single sequence edge, and to a single concordant edge as well, but it may connect to multiple discordant edges. The source nodes *s* and *t* connect to the canonically smallest and canonically largest coordinate on the reference genome from a collection of consecutive intervals connected by concordant edges, or a sequence edge that is only connected to another sequence edge with smaller CN by concordant edges and therefore is deemed to violate the balanced CN constraint without the source connections. Edges connected to source nodes are treated as discordant edges. For an example of a breakpoint graph constructed by CoRAL from the ecDNA in Figure 1A, see Figure 1B. We denote a maximal collection of genomic intervals connected by concordant edges as an *amplified interval* and denote the union of all amplified intervals and their (discordant) connections as an *amplicon*. Note that a tumor sample could contain multiple amplicons whose intervals are nonintersecting. CoRAL constructs a distinct breakpoint graph for each amplicon.

### Breakpoint graph construction with CoRAL

To build the breakpoint graph for an amplicon, CoRAL first determines all amplified intervals included in the amplicon. CoRAL requires *seed amplified intervals* (Supplemental Fig. S11A) as a starting point to search for all connected amplified intervals contained in an amplicon. The seed amplified intervals can be derived from whole-genome CNV calls (e.g., with third-party tools like CNVkit) (Talevich et al. 2016) of mapped long reads. From the CNV calls, we select the genomic segments adjacent to each other with a minimum threshold of copy number as well as the aggregated size as seed intervals (Supplemental Methods S2).

With the seed amplified intervals, CoRAL searches for amplified intervals connected to seed intervals (by discordant edges) using a breadth first search (BFS). For BFS, CoRAL maintains a list, I, of amplified intervals in all amplicons it explored or discovered so far, initialized as the list of seed intervals, and a set, E, representing the connections between amplified intervals through discordant edges. Each pair of intervals $\left(a_{i},\;a_{j})\in E\;(i\leq j\right)$ is labeled by the breakpoints connecting two loci within the intervals *a*_*i*_ and *a*_*j*_, respectively. The main iteration explores the next unvisited interval *a*_*i*_ in I, indicating a new amplicon (connected component), until all amplified intervals in I are visited. Let *L* be a priority queue used in the interval search starting from *a*_*i*_, which is initialized with a single element *a*_*i*_. Each step of the interval search pops the first interval *a*_0_ in *L* and extracts all breakpoints supported by chimeric alignments connecting a locus within *a*_0_ to another locus on the reference genome. These breakpoints are greedily clustered (with the procedure described in Supplemental Methods S2), and the new locus *l* determined by a cluster $bp_{a_{0},l}$ of breakpoints of the size at least of haploid coverage is chosen to be further explored (Supplemental Fig. S11B). If the new locus falls into an existing interval $a_{e}\in I$, then mark interval *a*_*e*_ as visited, augment the label set of (*a*_0_, *a*_*e*_) with $bp_{a_{0},l}$, and only append *a*_*e*_ to *L* if it was not previously visited. Otherwise, CoRAL will extend *l* to a new amplified interval including *l*, depending on whether *l* is amplified from the CNV calls. If *l* is amplified, CoRAL will append the new interval *a*_*n*_ = [chr_*l*_, max(*s*_*l*_ − δ*, l* − Δ), min(*e*_*l*_ + δ, *l* + Δ)] to both *L* and I, where *s*_*l*_ and *e*_*l*_ are the start and end coordinate of the amplified CN segments including *l* in CNV calls. If *l* is not amplified, CoRAL will append the new interval *a*_*n*_ = [chr_*l*_, *l* − δ, *l* + δ] to *L* and I. In either case, CoRAL also labels the connection (*a*_0_, *a*_*n*_) with $\left\{bp_{a_{0},l}\right\}$ and adds it to E. The amplified interval search starting from *a*_*i*_ is repeated until *L* becomes empty. A pseudocode of the above procedure, as well as the selection of Δ and δ, is discussed in detail in Supplemental Methods S2.

At the end of interval search, all intervals I are visited, and each connected component of amplified intervals by breakpoint edges with sufficient support of long reads forms an amplicon (Supplemental Fig. S11C). After BFS, CoRAL postprocesses the amplified intervals discovered in I by merging (1) adjacent (in CNV calls) or overlapping intervals or (2) intervals on the same chromosome that are not adjacent but have close (i.e., within ≤2δ–bp vicinity) breakpoint connections. Two intervals belonging to different amplicons are brought into the same amplicon after merging. CoRAL will then search for breakpoints within a single (merged) amplified interval (Supplemental Fig. S11D). Finally, CoRAL builds the actual breakpoint graph for each amplicon. It will split each amplification interval into sequence edges if there are breakpoint edges connecting to the middle of that interval and will add concordant edges connecting two adjacent sequence edges on the reference genome (Supplemental Fig. S11E).

### CN assignment

Once the graph structure G is fixed, CoRAL recomputes the CN for each edge in G (Supplemental Fig. S11E), as the initial CNV calls used for amplified interval search may not follow the balance requirement (Equation 1), and they do not account for concordant and discordant edges. Let the diploid long-read coverage be θ. CoRAL assumes that the majority of the donor genome is not amplified and estimates θ as the coverage on the 40th percentile of CN segments sorted by their initial CNV calls. Given θ, CoRAL models the total number of nucleotides on each sequence edge (*u*, *v*) ∈ *E*_*s*_ as a normal distribution with mean and variance θ · CN(*u*, *v*) · *l*(*u*, *v*), where *l*(*u*, *v*) denotes the length (in base pairs) of the sequence edge, and the number of reads supporting each concordant and discordant edge $(u,v)\in E_{c}\cup E_{d}$ as a Poisson with mean θ · CN(*u*, *v*). To estimate CN, CoRAL computes the maximum likelihood of CN using the joint distribution of observed number of nucleotides on each sequence edge and the observed read counts on each concordant/discordant edge with the constraint that CN is balanced (Supplemental Methods S3). The optimization problem was solved using CVXOPT package.

### Cycle extraction

We are interested in paths and cycles that alternate between sequence and breakpoint (i.e., concordant or discordant) edges; thus, by definition, if the path contains node *s* (respectively, *t*), it must be the first (respectively, last) node in the path. Define an *alternating sequence* of nodes as a sequence *v*_1_, *v*_2_, … , *v*_*w*_, where for all 1 ≤ *i* < *w*, (*v*_*i*_, *v*_*i*+1_) ∈ *E* and the edges alternate between sequence and breakpoint edges. Define a *walk* in G as an alternating sequence *v*_1_, *v*_2_, … , *v*_*w*_, where *v*_1_ = *s*, *v*_*w*_ = *t*. A *path* is a walk with no node repeated (*v*_*i*_ = *v*_*j*_ ⇔ *i* = *j*). A *cyclic walk* or *cycle* is an alternating sequence *v*_1_, *v*_2_, … , *v*_*w*_ of nodes where *v*_1_ = *v*_*w*_ ≠ *s*, *t*. The cycle is simple if no node except the first/last one is repeated.

The amplicon encoded by G is composed of a superposition of cycles and walks with high copy numbers. For all sequence edges (*u*, *v*) ∈ *E*_*s*_, define the *length-weighted copy number* using C_*l*_(*u*, *v*) = CN(*u*, *v*) · *l*(*u*, *v*). Similarly, for graph G,

(2) $$C_{l}(G)=\underset{(u,v)\in E_{s}}{\sum }C_{l}(u,v).$$

Our goal is to identify a minimum number of cycles and walks (denoted as *W*_*i*_), each associated with a positive weight, corresponding to the copy number (based on the assumption of uniform coverage) (see Supplemental Fig. S12), so that the sum of weights on all edges in all walks composes a large fraction of $C_{l}(G)$. Furthermore, the long reads that span multiple (at least two) breakpoints in G also provide us with a collection of subwalks $P=\{\;p_{1},\ldots ,\;p_{m}\}$, and the reconstructed walks must simultaneously be consistent with a large fraction of these subwalks (Supplemental Methods S4).

The complexity of cycle extraction and rationale for CoRAL's optimization procedure can be shown through an example illustration (Supplemental Fig. S13). The breakpoint graph in Supplemental Figure S13A consists of segments A, B, and C assumed for simplicity to be of equal length. The optimization in CoRAL will decompose it into a single cycle of copy number 50, with a duplication of segment B (right panel). The decomposition is also supported by the subwalk constraint given by the long read that connects segments A, B, and C. Even if the long read were not present, this cycle is still a parsimonious solution compared to an alternative decomposition with two cycles (one containing A and B, the other containing B and C).

Supplemental Figure S13B has a similar graph structure but with different copy numbers on segments. The best decomposition is given by one cycle containing A and B with copy number 80 and a second cycle containing A, B (two copies), and C with copy number 10. The total length-weighted copy number of the graph is 200 (assuming segments of length one). Cycle 1 explains 80% and cycle 2 explains 20% of the copy number. Other decompositions of cycles are indeed possible. For example, if the subwalk constraint given by the long read were not present, an alternative decomposition with 90 copies of cycle 1 and 10 copies of a different cycle 2 containing only segments B and C would also explain all length-weighted copy numbers in the graph. On the other hand, a more parsimonious decomposition into one single cycle with copy number 10, with segment A repeating nine times and segment B repeating 10 times is not allowed because it violates the upper bound on the multiplicity of segments in the cycle (see auxiliary constraint 3 in the MIQCP formulation below). For the same reason, the decomposition into one single cycle is not allowed in Supplemental Figure S13C.

We resolve the multiobjective challenge using mixed integer quadratically constrained programing (MIQCP). The MIQCP works with two parameters: α as the minimum fraction of length-weighted copy number explained, and β as the minimum fraction of path constraints satisfied. Additionally, parameter *k* denoting the maximum number of cycles/walks allowed is learned starting with *k* = 10, according to two modes. In the **full** mode (MIQCP-full) described below, the MIQCP attempts a solution with at most *k* walks that satisfy other constraints or that returns “infeasible.” The value of *k* is doubled until feasibility is reached or *k* > |*E*|. The **greedy mode** is described later. We implement both quadratic programs through the Python 3 interface of Gurobi 10.0.1.

MIQCP-full utilizes the following **key** variables:

- *w*_*i*_ ∈ ℚ > 0 denotes the copy number for walk *W*_*i*_ (1 ≤ *i* ≤ *k*), and *z*_*i*_ ∈ {0, 1} indicates if *w*_*i*_ > 0.
- *x*_*uvi*_ ∈ ℤ ≥ 0 represents the number of times walk *W*_*i*_ traverses (*u*, *v*) for each edge (*u*, *v*) ∈ *E* and 1 ≤ *i* ≤ *k*.
- *P*_*j*_ ∈ {0, 1} indicates if subwalk constraint *p*_*j*_ is satisfied for 1 ≤ *j* ≤ *m*.

The MIQCP(*k*, α, β) objective is given by

(3) $$\min\underset{\mathrm{\#walks}}{\underbrace{\overset{k}{\underset{i=1}{\sum }}z_{i}}}-\underset{\mathrm{fraction}\;\mathrm{of}\;C_{l}(G)\;\mathrm{explained}}{\underbrace{\frac{1}{C_{l}(G)}\overset{k}{\underset{i=1}{\sum }}\underset{(u,v)\in W_{i}\cap E_{s}}{\sum }w_{i}\cdot x_{uvi}\cdot l(u,v)}}-\underset{\mathrm{fraction}\;\;\mathrm{of}\;\mathrm{subwalks}\;\mathrm{satisfied}}{\underbrace{\frac{1}{m}\overset{m}{\underset{\;j=1}{\sum }}P_{j}}},$$

subject to the constraints

(4) $$w_{i}\leq z_{i}\cdot C_{l}(G),$$

(5) $$\overset{k}{\underset{i=1}{\sum }}\underset{(u,v)\in W_{i}\cap E_{s}}{\sum }w_{i}\cdot x_{uvi}\cdot l(u,v)\geq \alpha \cdot C_{l}(G),$$

(6) $$\overset{m}{\underset{\;j=1}{\sum }}P_{j}\geq \text{β}\cdot m.$$

Equation 4 ensures that *w*_*i*_ = 0 if $z_{i}=0,\;\forall i=1,\ldots ,k$, and Equations 5 and 6 ensure that minimum fractions of the length-weighted copy number and subwalk constraints are satisfied. The unsatisfied fractions also contribute a small amount to the MIQCP objective. To ensure that cycles and walks have their nodes connected and satisfy the alternating-edge structure, we must satisfy several auxiliary constraints, enumerated below, with details in the Supplemental Methods (Equations S4.6–S4.23):

1. Each *W*_*i*_ should form a valid walk of alternating sequence and breakpoint (i.e., concordant or discordant) edges.
2. The total CN of all cycles/walks passing through an edge (*u*, *v*) ∈ *E* is at most C_*l*_(*u*, *v*).
3. We require that each cycle/walk traverses through a discordant edge (*u*, *v*) at most *R*(*u*, *v*) times. By default, the value of *R*(*u*, *v*) is estimated for each discordant edge (*u*, *v*) ∈ *E*_*d*_ based on the number of (long) reads supporting that edge (for details, see Supplemental Methods S4).
4. Each walk *W*_*i*_ (if *z*_*i*_ > 0) either forms a cycle starting at node *v*_1_ ≠ *s, t* or starts at *s* and ends at *t*. If *W*_*i*_ forms a cycle, we require that the concordant or discordant edge connected to *v*_1_ occurs only once in the cycle.
5. *x*_*uvi*_ and *z*_*i*_ are consistent. *z*_*i*_ = 1 ⇔ *x*_*uvi*_ > 0 for some (*u*, *v*) ∈ *E*.
6. **Connectivity.** We use auxiliary variables to encode the “discovery order” of the nodes in walk *W*_*i*_. These variables number the nodes from “1” for the start node and increment by one for each subsequent node in the cycle/walk.
7. **Subwalk constraints.** We enforce a weak constraint by requiring each walk $\;p_{j}\in P$ to be present as a subgraph of the graph induced by some walk *W*_*i*_.

### MIQCP-greedy(α, β, γ, ɛ)

For a large graph (e.g., |*E*| > 100), MIQCP-full could be resource intensive. Therefore, we also implemented an MIQCP with the additional parameters γ and ɛ, but not *k*, that identifies only a single walk maximizing the copy number and additional subwalk constraints satisfied, with parameter γ controlling the weight of the two objectives. Let

$$\bar{P}=\{j\;|\;\mathrm{path}\;\;p_{j}\;\mathrm{is}\;\mathrm{not}\;\mathrm{satisfied}\;\mathrm{by}\;\mathrm{any}\;\mathrm{previously}\;\mathrm{selected}\;\mathrm{walk}\}.$$

Then, the greedy MIQCP objective to identify the next walk *W*_*i*_ is given by

(7) $$\max\underset{(u,v)\in W_{i}\cap E_{s}}{\sum }w\cdot x_{uv}\cdot l(u,v)+\gamma \cdot \underset{\;j\in \bar{P}}{\sum }P_{j}.\;$$

Each time a new walk is computed, its copy number is removed for all edges it passed through, and $\bar{P}$ is updated. The procedure is repeated until either $\alpha \cdot C_{l}(G)$ copy numbers and β · *m* subwalk constraints are explained by the currently selected walks or the copy number of next walk is less than $\varepsilon \cdot C_{l}(G)$ for parameter ɛ. We empirically set $\gamma =0.01C_{l}(\bar{G})/|\bar{P}|$, where $\bar{G}$ denotes the remaining length-weighted copy number of G after removing the copy numbers from the last walk, and ɛ = 0.005. The greedy MIQCP is solved using the same set of auxiliary constraints as before.

### Implementation details

In practice, if G has |*E*| > 100 edges, we use the iterative greedy MIQCP, until either 90% of length-weighted copy number is removed from the graph or the length-weighted copy number of the next cycle is <1% of the total amount in the breakpoint graph. Otherwise, we run full-MIQCP with α = 0.9, β = 0.9. Initially, *k* = 10, and it is doubled until a feasible solution is reached. If doubling the number of cycles/paths leads to more than 10,000 variables in the integer program, we switch to greedy-MIQCP. CoRAL provides users an option to postprocess the greedy-MIQCP solutions with full MIQCP with $\alpha =\min(0.9,\;1-C_{l}(\bar{G})/C_{l}(G))$, $\beta =\min(0.9,\;1-|\bar{P}|/|P|)$.

## Data access

All raw and processed sequencing data generated in this study have been submitted to the NCBI BioProject database (https://www.ncbi.nlm.nih.gov/bioproject/) under accession number PRJNA1110283. Other short-read and long-read sequencing data used in this study can be found in Supplemental Methods S6. The version of CoRAL used in the presented analysis is included as Supplemental Code. An up-to-date version of CoRAL is available on GitHub (https://github.com/AmpliconSuite/CoRAL).

## Supplementary Material

Supplement 1

Supplement 2

Supplement 3

Supplement 4

Supplement 5

Supplement 6

Supplement 7
