# Supplementary material for: CoRAL accurately resolves extrachromosomal DNA genome structures with long-read sequencing
Source: Genome Res. 2024 Sep;34(9):1344–54. doi: 10.1101/gr.279131.124 (PMC11529860; doi:10.1101/gr.279131.124)
Supplement: Supplement 5 [file Supplemental_Figures.pdf]

Supplemental Figures for CoRAL accurately resolves extrachromosomal DNA genome structures with long-read sequencing

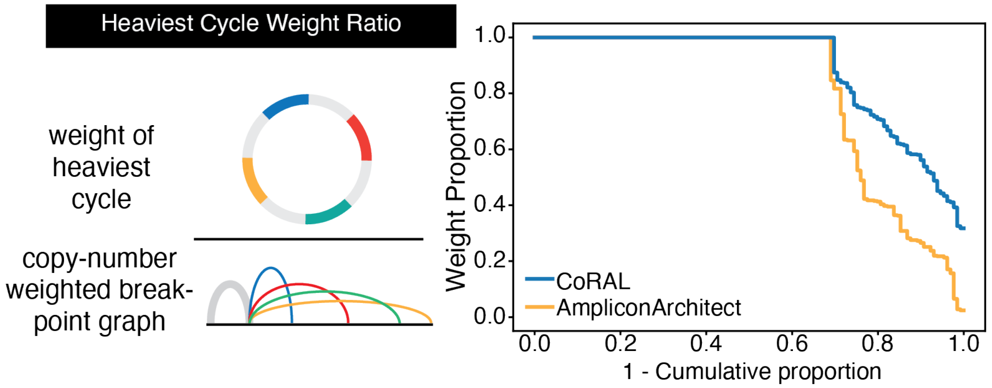

Supplemental Figure S1: **Fraction of length-weighted copy numbers given by the heaviest cycle from AA and CoRAL reconstructions.** Fraction of length-weighted copy numbers given by the heaviest cycle over the total length-weighted copy numbers in the inferred breakpoint graph (i.e., "Heaviest cycle weight ratio" below) is reported across all simulated amplicons.

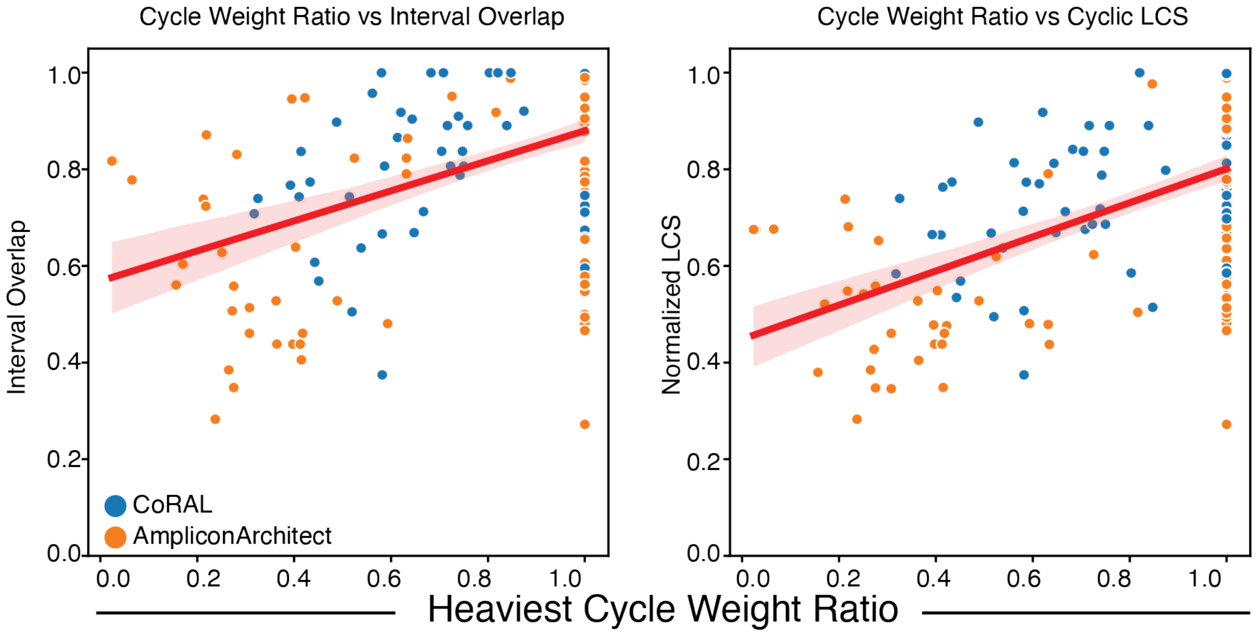

Supplemental Figure S2: **Comparison of the cycle weight ratio vs accuracy measures of simulated benchmarks.**

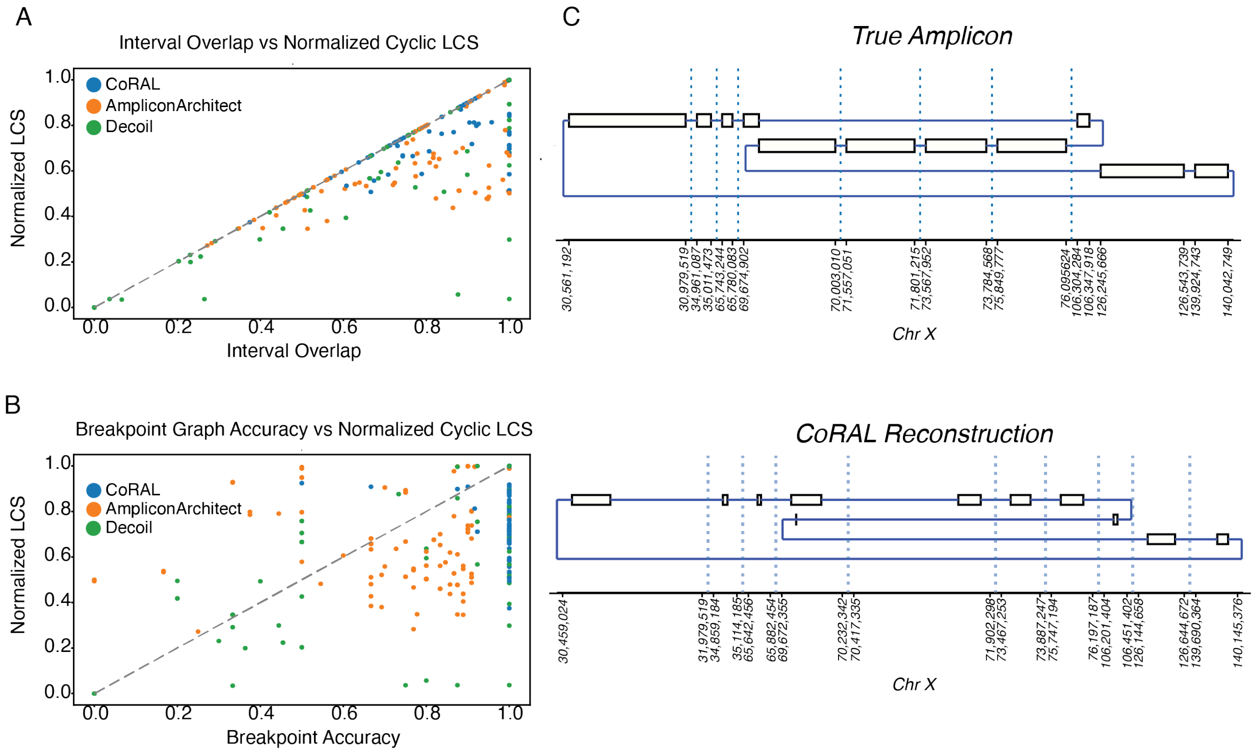

Supplemental Figure S3: **Reconstructions with perfect overlap but imperfect ordering.** Evaluation of the Normalized Cyclic LCS (which captures ordering of intervals) vs the interval overlap (A) and breakpoint accuracy (B). An example CoRAL reconstruction with an interval overlap of 0.99 but a normalized cyclic LCS of 0.51 due to an incorrectly inverted set of intervals.

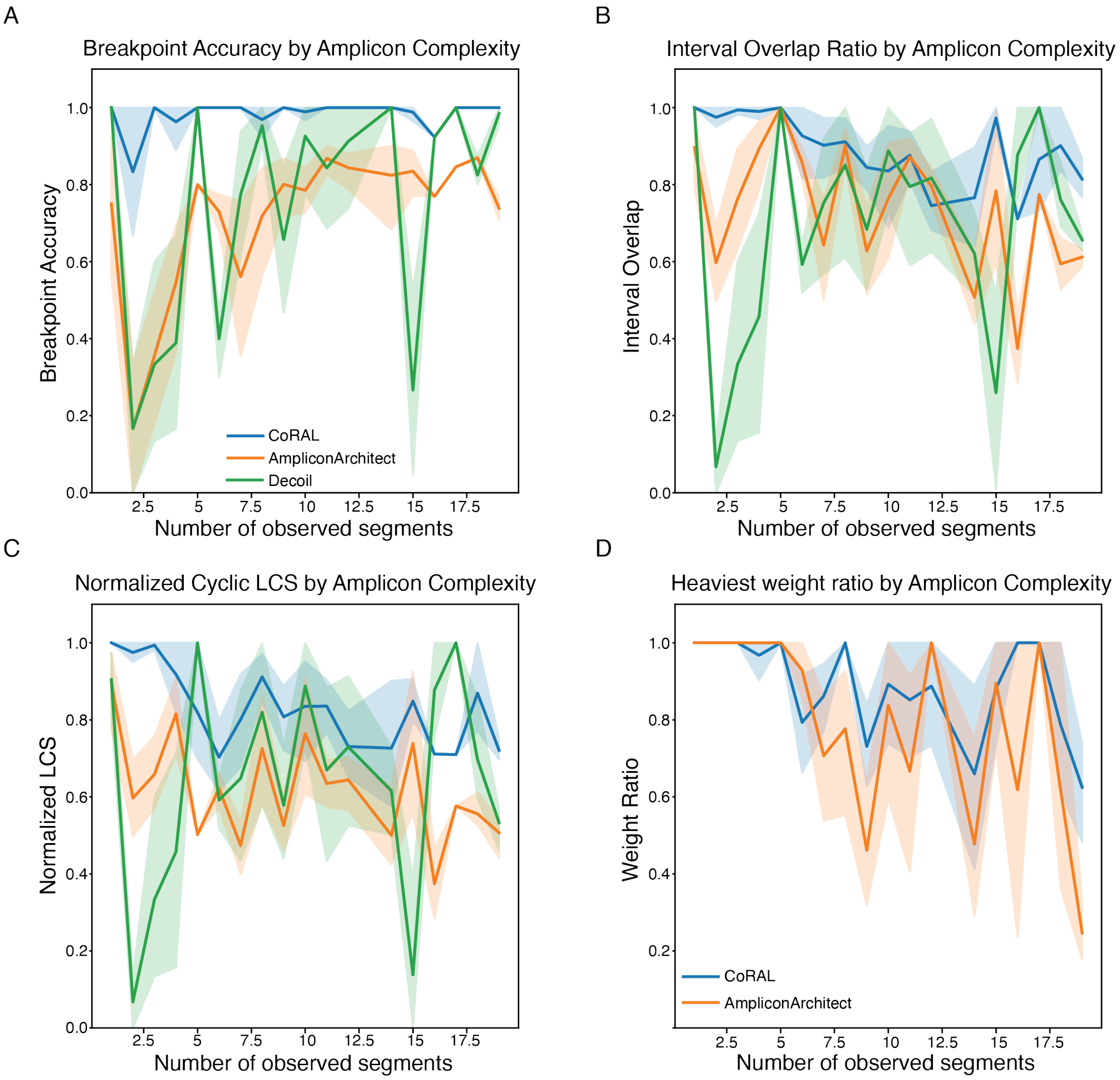

Supplemental Figure S4: **Performance on simulated data separated by number of genome segments in the true amplicon.** Performance of CoRAL, AmpliconArchitect, and Decoil (A-C only) on simulated amplicons as a function of the number of simulated genome segments, capturing breakpoint accuracy (A), interval overlap ratio (B), normalized cyclic longest-common substring (LCS; C), and heaviest cycle weight ratio (D).

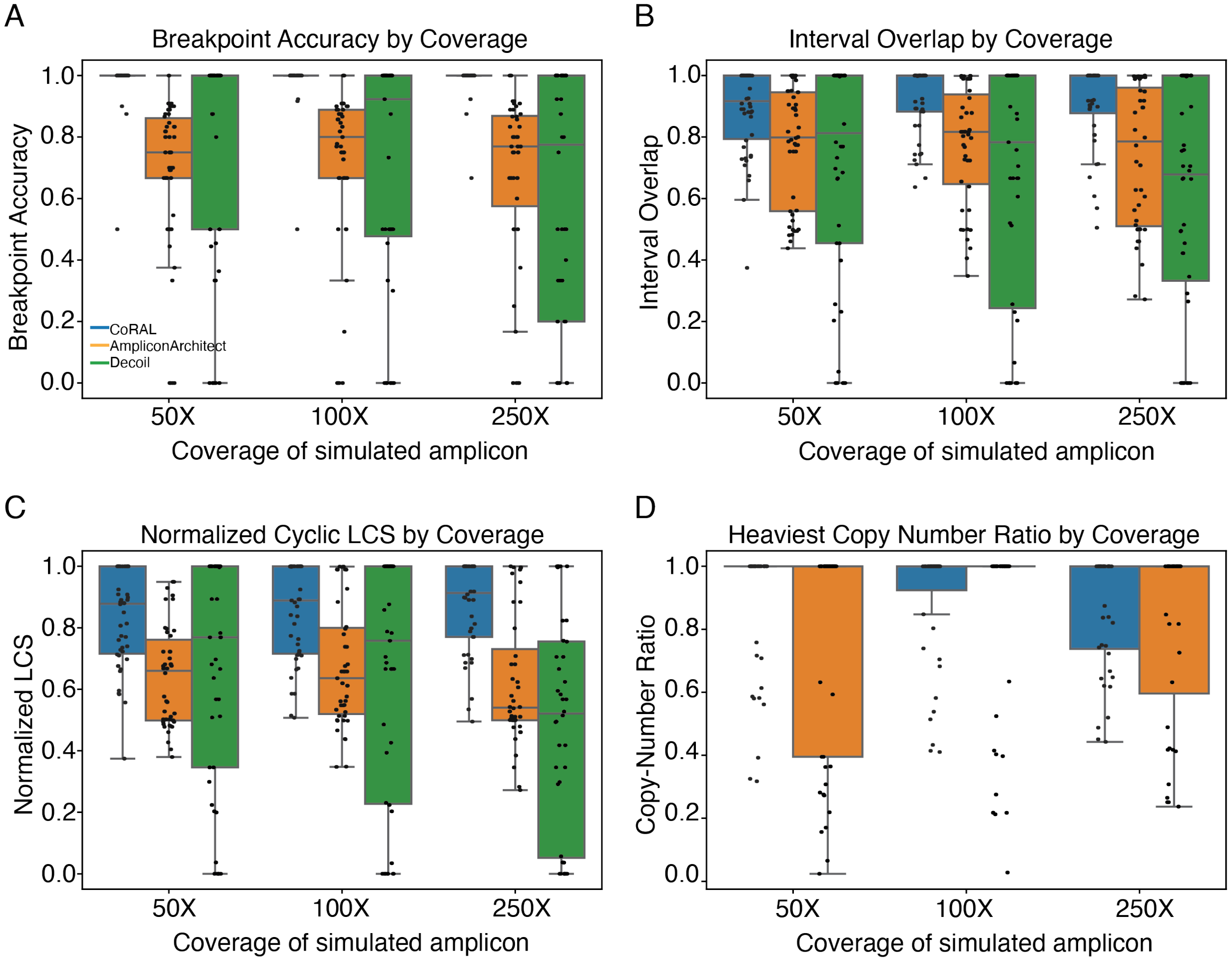

Supplemental Figure S5: **Accuracy of simulations by simulated amplicon coverage.** Breakpoint accuracy (A), interval overlap ratio (B), normalized cyclic longest-common substring (LCS; C), and heaviest cycle weight ratio (D) for simulations broken down by simulated amplicon coverage.

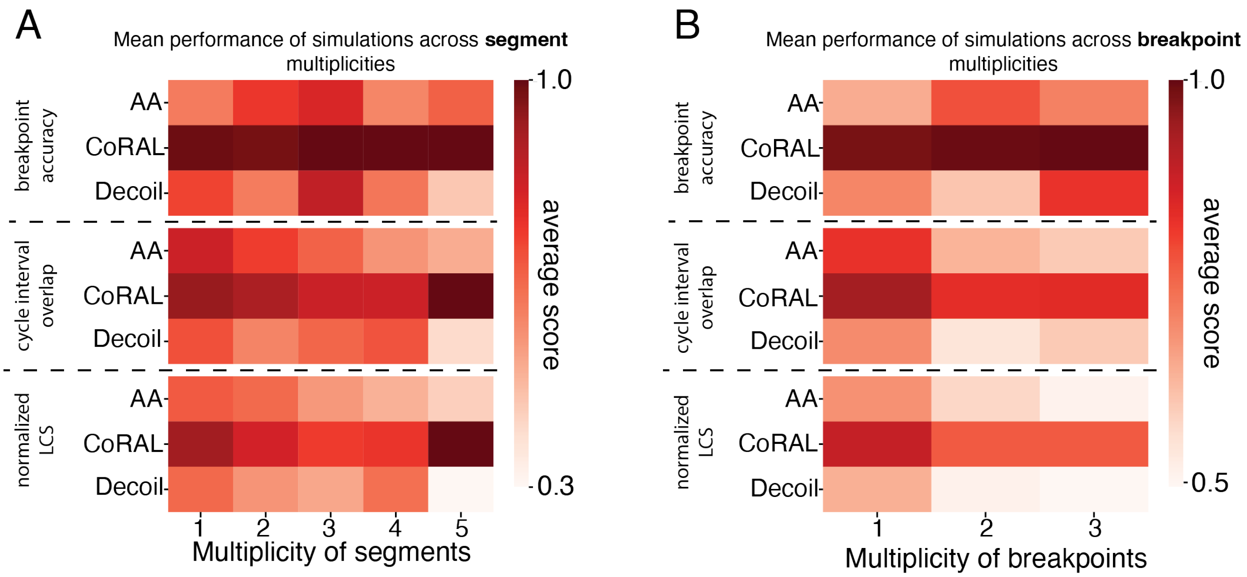

Supplemental Figure S6: **Accuracy of simulations by multiplicity of segments and breakpoints in amplicon.** Breakpoint accuracy, interval overlap ratio, and normalized cyclic longest-common substring (LCS) for simulations reported as a function of (A) largest multiplicity of segments, and (B) largest multiplicity of breakpoints. Values in heatmaps correspond to the mean performance across replicates.

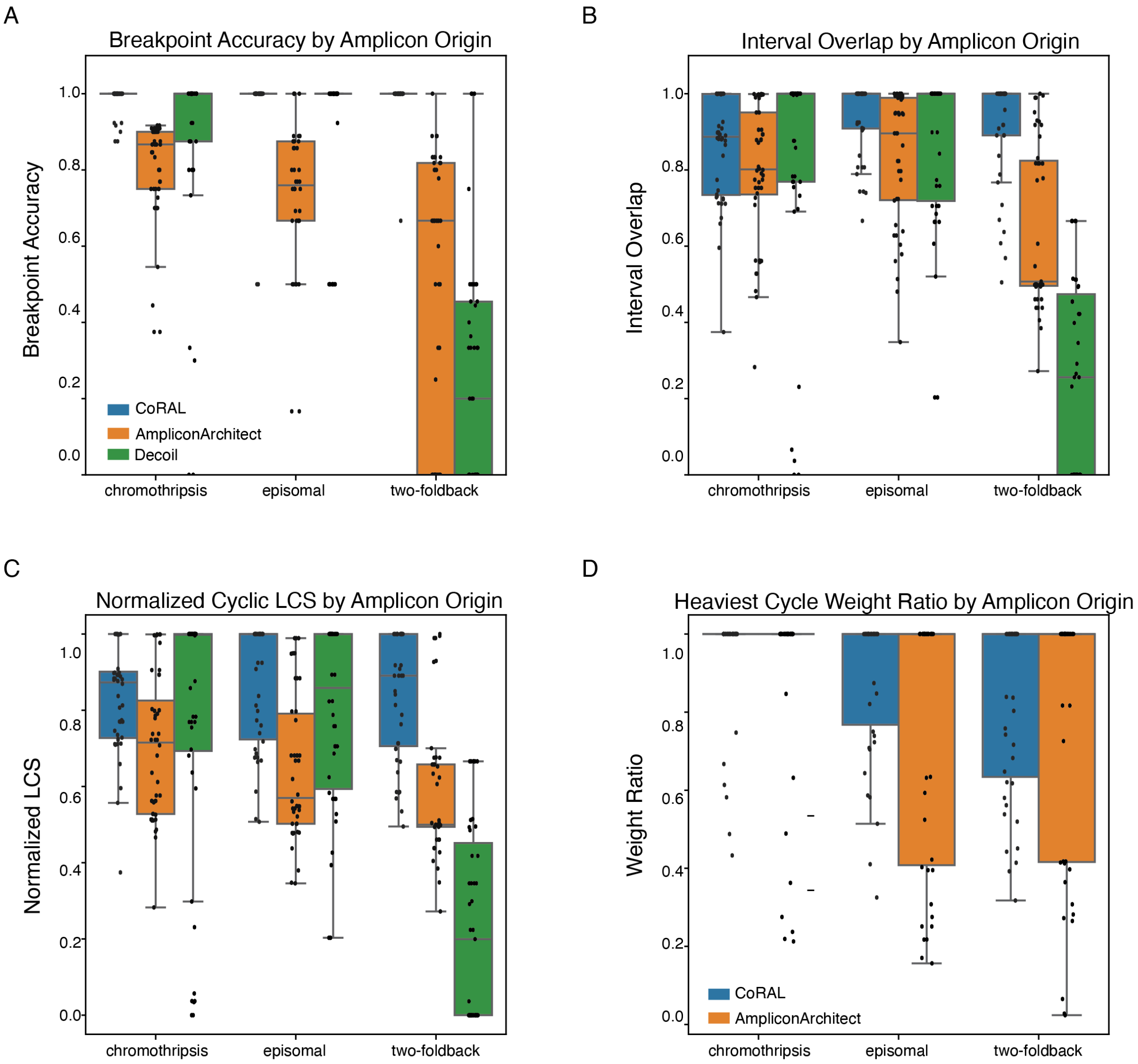

Supplemental Figure S7: **Accuracy of simulations by simulated amplicon origin.** Breakpoint accuracy (A), interval overlap ratio (B), normalized cyclic longest-common substring (LCS; C), and heaviest cycle weight ratio (D) for simulations broken down by the origin mechanism of simulated amplicon.

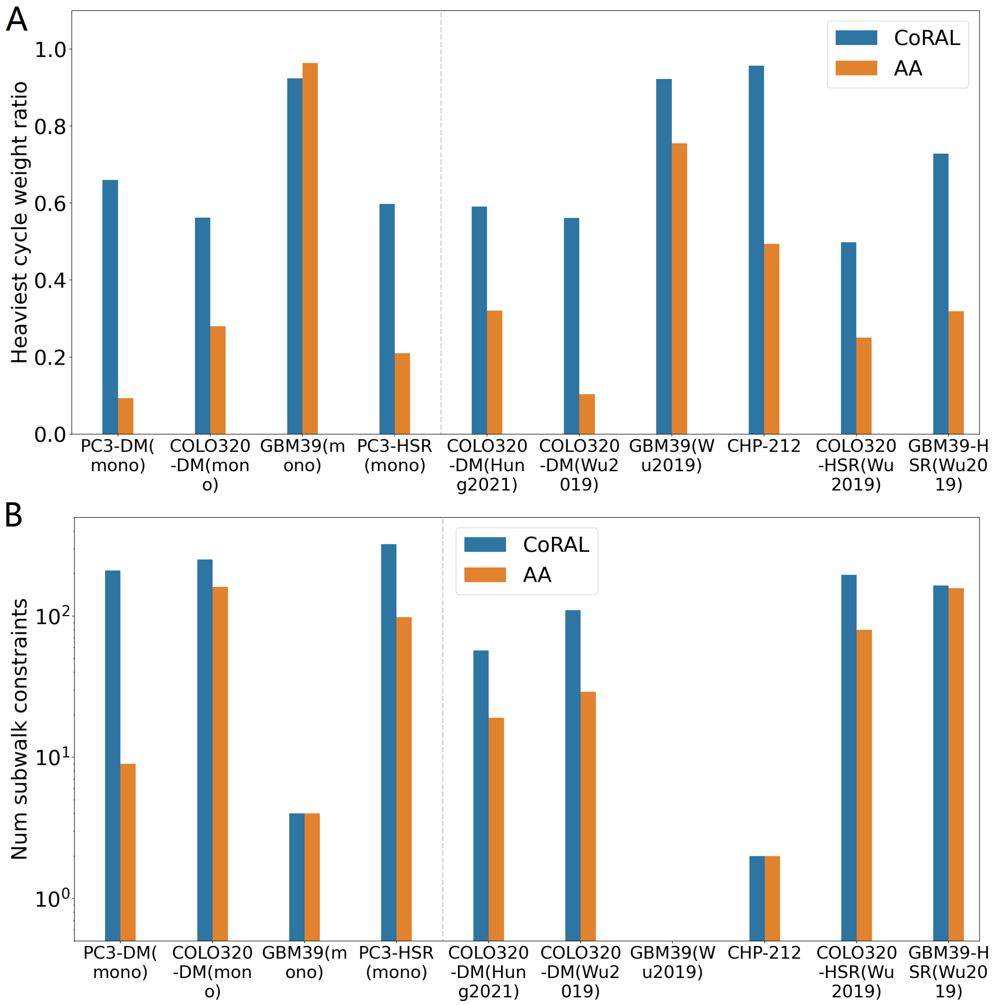

Supplemental Figure S8: **Amplicon reconstruction performance in cell lines, with a single heaviest cycle.** (A) Heaviest cycle weight ratio from a single heaviest cycle (i.e., $k = 1$) reported by CoRAL and AA; (B) number of subwalk constraints satisfied by the heaviest cycle reported by CoRAL and AA, in cell lines.

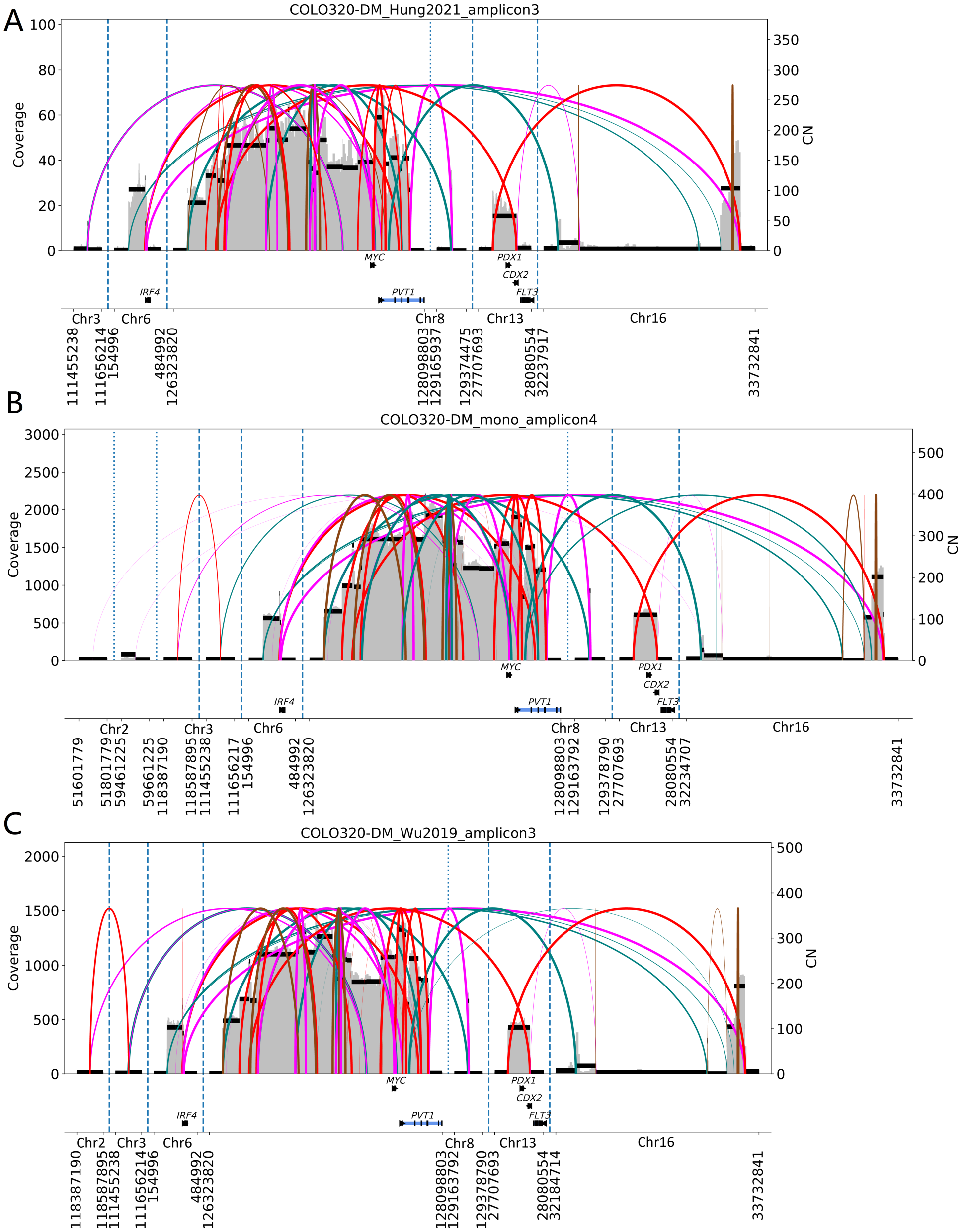

Supplemental Figure S9: **Breakpoint graph reconstructed by CoRAL from the three COLO320-DM samples.** (A) COLO320-DM (Hung 2021) breakpoint graph reconstructed by CoRAL. (B) COLO320-DM (mono) breakpoint graph reconstructed by CoRAL. (C) COLO320-DM (Wu 2019) breakpoint graph reconstructed by CoRAL.

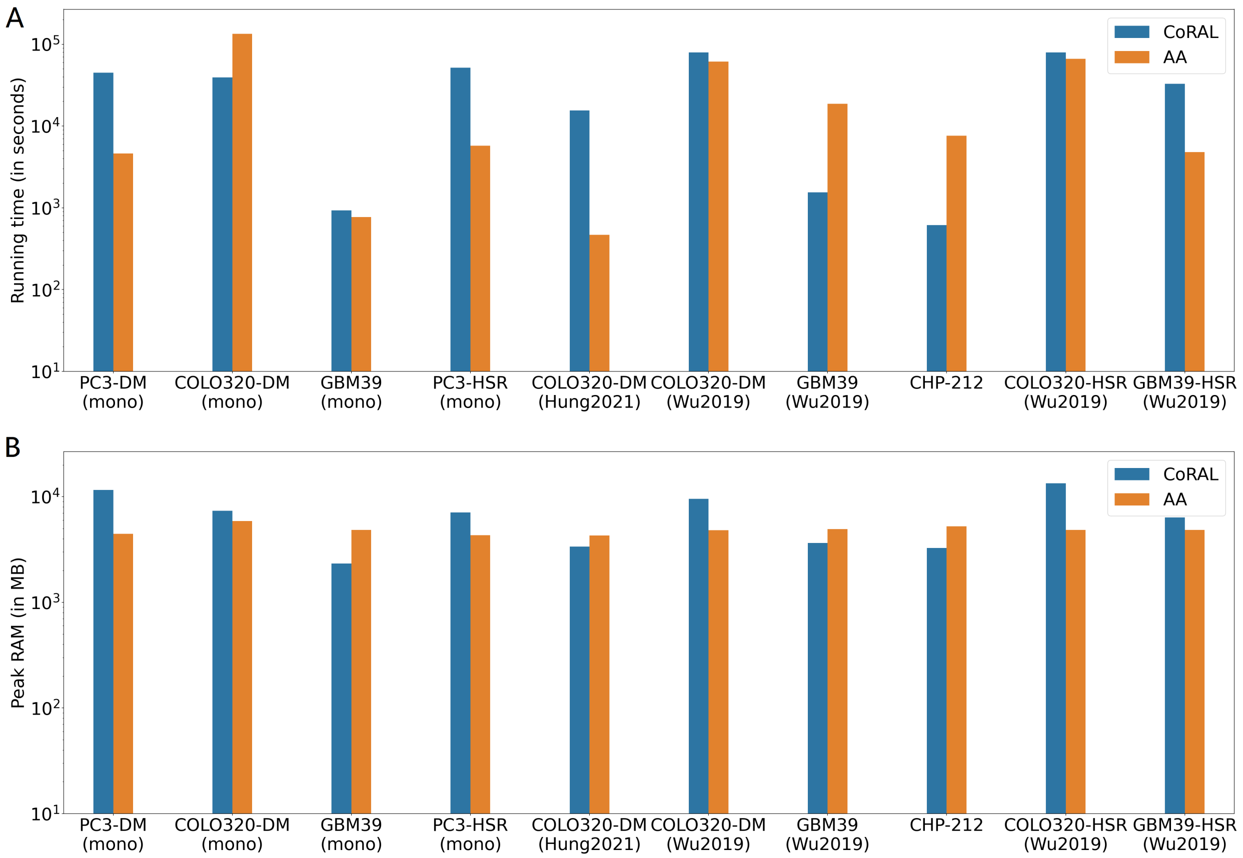

Supplemental Figure S10: **Running time and memory comparison between CoRAL and AA.** (A) Running time, in seconds; (B) Peak RAM usage, in Megabytes, to reconstruct all amplicons (and cycles) in each sample.

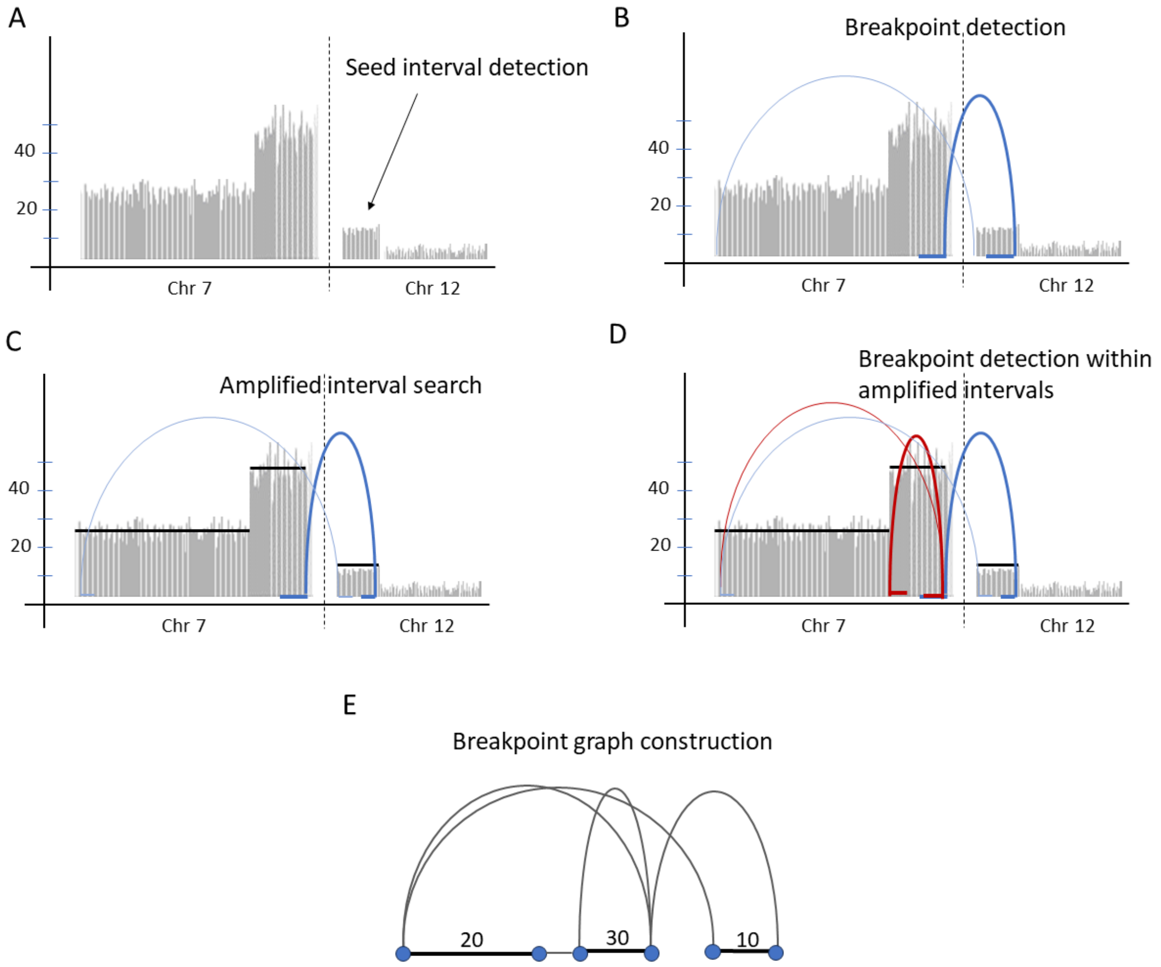

Supplemental Figure S11: **Illustration of amplified interval search and breakpoint graph construction.** (A) CoRAL starts with seed amplified intervals to search for all connected amplified intervals contained in an amplicon. (B) CoRAL explores new amplified intervals connected to seed intervals through breakpoints. (C, D) CoRAL first searches for all amplified intervals constituting an amplicon and then detects additional breakpoints within each amplified interval. (E) Finally, CoRAL builds a breakpoint graph with the list of amplified intervals, splitting the intervals at breakpoint junctions.

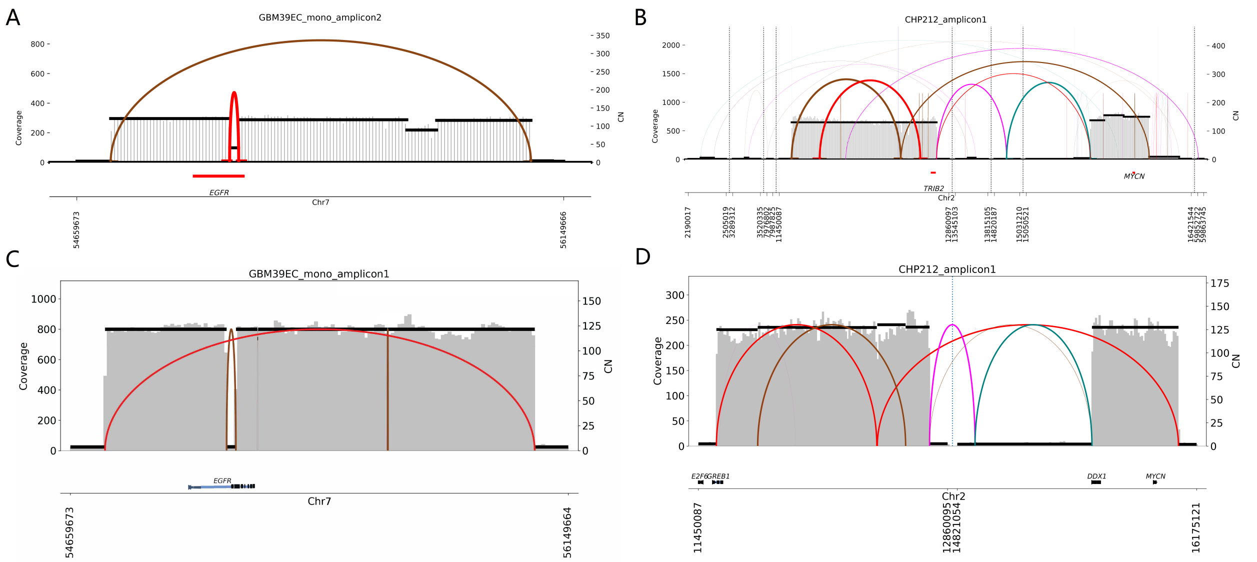

Supplemental Figure S12: **Breakpoint graph reconstructed from cell lines indicating uniform coverage on ecDNA.** Breakpoint graph reconstructed from (A) GBM39 (mono) short reads; (B) GBM39 (mono) long reads; (C) CHP-212 short reads; (D) CHP-212 long reads. Gray vertical bars indicate (short/long reads) coverage and black horizontal lines indicate predicted CN for each sequence edge in the graph.

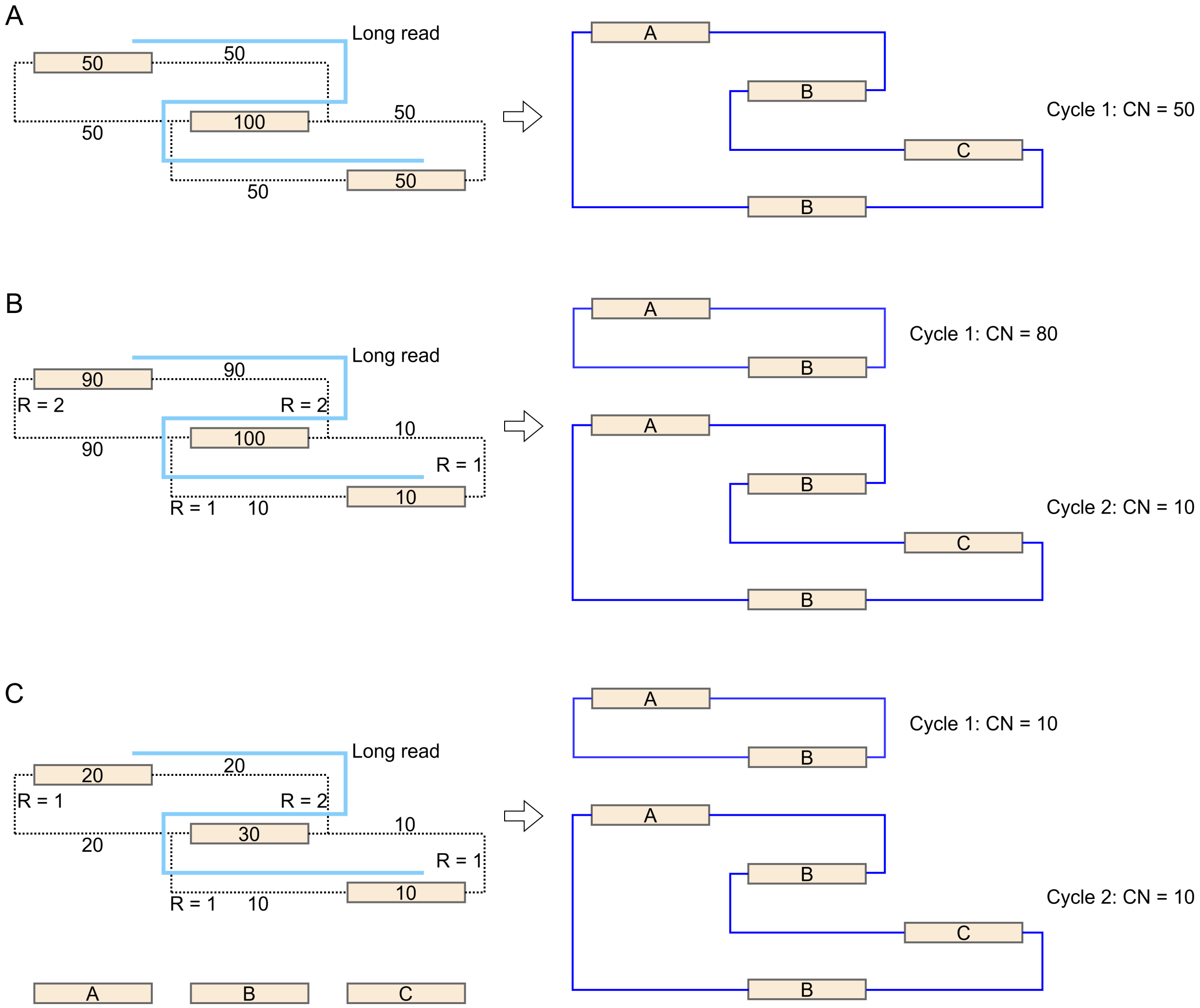

Supplemental Figure S13: **Cycle decomposition with subwalk constraints.** (A) A single cycle with copy number 50 containing 2 copies of segment B and 1 each of segments A and C accounts for 100% of the copy number in the breakpoint graph. It also satisfies the subwalk constraint given by the long read. (B) The copy number of the breakpoint graph is explained by a minimum of 2 cycles, with copy numbers 80 and 10 respectively, due to the number of times each discordant edge can be traversed in a cycle (marked on the discordant edge as $R = 1$ or $R = 2$). (C) The copy number of the breakpoint graph is explained by a minimum of 2 cycles, with copy numbers 10 and 10 respectively, again due to a limit on the number of times each discordant edge can be traversed in a cycle.
