# Supplementary material for: CoRAL accurately resolves extrachromosomal DNA genome structures with long-read sequencing
Source: Genome Res. 2024 Sep;34(9):1344–54. doi: 10.1101/gr.279131.124 (PMC11529860; doi:10.1101/gr.279131.124)
Supplement: Supplement 6 [file Supplemental_Methods.pdf]

Supplemental Methods for CoRAL accurately resolves extrachromosomal DNA genome structures with long-read sequencing

S1. Preliminaries

We begin by offering a set of definitions that are used throughout this paper.

Reference Genome. A **reference genome** is a set of R strings $\mathcal{RG} = \{s_1, \cdots, s_R\}$ representing the chromosomes in a genome. For example, the human genome $\mathcal{RG}$ is comprised of $R = 24$ strings typically labeled as $Chr1, \cdots, Chr22, ChrX$, and $ChrY$. We denote the length of string s_i with $|s_i|$. At times we call strings in $\mathcal{RG}$ **chromosomes** and enforce a total order $\prec$ on the chromosomes (e.g., the human genome string $Chr2$ is smaller than $Chr3$ and thus appears before it). A **genomic interval** $s_i[l, r]$ is a sequence of nucleotides in string s_i starting at position l and ending at position r (both inclusive) where $1 \leq l \leq r \leq |s_i|$. At times, we use $s_i[l, \ldots]$ to denote an interval starting at position l and $s_i[\ldots, r]$ as a interval ending at position r.

Breakpoint. A **breakpoint** describes a junction between two genomic intervals in the reference genome set. We describe a breakpoint bp by a pair of triplets $(s_1, p_1, o_1, s_2, p_2, o_2)$ indicating the two ends of the breakpoint:

- chromosomes in the reference genome (s_1, s_2) (both $s_1, s_2 \in \mathcal{RG}$);
- starting or ending positions of each interval (p_1, p_2); and
- and orientations (o_1, o_2) (both $o_1, o_2 \in \{+, -\}$)

The positions p_1 and p_2 denote starting or ending positions, depending on the orientation. Specifically:

- if $o_1 = +$ and $o_2 = +$, b describes a junction between genomic intervals $s_1[\ldots, p_1]$ and $s_2[\ldots, p_2]$.
- if $o_1 = +$ and $o_2 = -$, b describes a junction between genomic intervals $s_1[\ldots, p_1]$ and $s_2[p_2, \ldots]$.
- if $o_1 = -$ and $o_2 = +$, b describes a junction between genomic intervals $s_1[p_1, \ldots]$ and $s_2[\ldots, p_2]$.
- if $o_1 = -$ and $o_2 = -$, b describes a junction between genomic intervals $s_1[p_1, \ldots]$ and $s_2[p_2, \ldots]$.

We note that $(s_1, p_1, o_1, s_2, p_2, o_2)$ and $(s_2, p_2, o_2, s_1, p_1, o_1)$ describe the same breakpoint. To facilitate comparisons over breakpoints, we therefore require either $s_1 \prec s_2$ or $s_1 = s_2$, $p_1 < p_2$. It is also possible, though rare, that a breakpoint connects $(s_1, p_1, +)$ to $(s_1, p_1, -)$, representing a single nucleotide duplication. As such we ignore these cases.

Breakpoint Graph. A **Breakpoint Graph** is a weighted, undirected graph $\mathcal{G} = (V, E = E_s \cup E_c \cup E_d, \text{CN})$ that contains information pertaining to breakpoints in a sample. We define the entities in this graph as follows:

V ($V \subseteq \mathcal{RG} \times \mathbb{N} \times \{+, -\}$) represents either the starting or ending position of a genomic interval (except the two special source nodes s and t, as described below);

E_s represents **sequence edges**, or connections between the starting and ending positions in a genomic interval;

E_c represents the **concordant edges** that connect two consecutive genomic intervals $s_i[\ldots, p]$ and $s_i[p+1, \ldots]$.

E_d represents the **discordant edges** connecting two nodes $v_1 = (s_1, p_1, o_1)$ and $v_2 = (s_2, p_2, o_2)$ where $s_1 \neq s_2$, or $|p_1 - p_2| > 1$, or $o_1 = o_2$. A discordant edge could also connect a node $v = (s_1, p_1, o_1)$ to itself - we call such discordant edges **foldbacks**. Foldback edges will introduce self-loops in a breakpoint graph. We sometimes refer to a **breakpoint edge** as either a concordant or discordant edge in the breakpoint graph, indicating a discontinuity on the reference genome.

CN CN maps each edge to a fractional **copy number**: $\text{CN} : E \rightarrow \mathbb{Q}+$.

Note that in a breakpoint graph, each node is connected to a single sequence edge, and a single concordant edge as well; but it may connect to multiple discordant edges. Similar to (Deshpande et al. 2019; Hadi et al. 2020; Aganezov and Raphael 2020), we require that the CN assignment is "balanced" for each sequence edge $(u, v) \in E_s$, i.e., the sum of CN values from the breakpoint edges connected to (u, v) equals to the CN of thos sequence edge. A node connected to a foldback edge receives two times the CN from the foldback edge it connects to when summing up CN values from discordant edges.

$$\sum_{(w,u)\in E_c\cup E_d} \text{CN}(w, u) = \text{CN}(u, v) = \sum_{(v,w)\in E_c\cup E_d} \text{CN}(v, w),\ \forall(u, v) \in E_s \quad \text{(S1.1)}$$

As per Amplicon Architect (AA) (Deshpande et al. 2019), **amplified intervals** (or **amplicon intervals**) describes a set $\mathcal{I} = \{(s_i, l_i, r_i)\}$ as the union of genome intervals represented by all sequence edges in a breakpoint graph - where l_i gives the smallest coordinate among a few sequence edges connected by concordant edges; and r_i gives the largest coordinate among a few sequence edges connected by concordant edges. Each string s_i in $\mathcal{RG}$ can include multiple, non-adjacent amplified intervals: for $(s_i, l_i, r_i), (s_i, l'_i, r'_i) \in I, l_i > r'_i + 1$ or $l'_i > r_i + 1$. The simplest non-empty breakpoint graph gives a single amplified interval, a single sequence edge, and no breakpoint (concordant/discordant) edges. We refer to an **amplicon** as the union of all amplified intervals and their (discordant) connections. In other words, an amplicon corresponds to a breakpoint graph. In a tumor sample there could be multiple amplicons whose intervals are non-intersecting.

In addition to the nodes representing the starting or ending position of a genomic interval (sequence edge), we introduce two artificial nodes, $s \in V$ and $t \in V$ in each breakpoint graph $\mathcal{G}$. They both connect to the end of each amplified interval, as well as a sequence edge which is only connected to another sequence edge with smaller CN by concordant edges, and therefore is deemed to violate the balanced CN constraint S1.1 without the source connections. Edges connected to source nodes are treated as discordant edges.

A **walk** in a breakpoint graph $\mathcal{G}$ is a sequence of nodes $v_1, v_2, \ldots, v_w$, where for all $1 \leq i < w$, $(v_i, v_{i+1}) \in E$, and the edges alternate between sequence and breakpoint edges (i.e. if $(v_i, v_{i+1}) \in E_s$ then $(v_{i-1}, v_i) \in E_c \cup E_d$ and $(v_{i+1}, v_{i+2}) \in E_c \cup E_d$). A *path* is a walk with no node repeated ($v_i = v_j \Leftrightarrow i = j$). An **$s, t$-walk/path** in a walk or path starts at s and ends at t. A **cycle** or **cyclic walk** is a walk where the first edge starts with and the last edge ends with the same node, i.e., $v_1 = v_w \neq s, t$. The cycle is **simple** if no node except the first/last one is repeated.

With these definitions, we say that a properly reconstructed breakpoint graph $\mathcal{G}$ represents a superimposition of all ecDNAs and rearranged genomes. These rearranged genomes are represented

by walks of alternating sequence edges and breakpoint edges. For example, an s,t-path of alternating sequence and breakpoint edges may represent a linear focal amplification; ecDNA form cycles of alternating sequence and breakpoint edges.

S2. Breakpoint Graph Reconstruction from Long Reads

Seed Interval Detection. CoRAL detects seed amplified intervals from whole genome CNV calls of mapped long reads (e.g., with third party tools like CNVkit (Talevich et al. 2016)). In fact, CNV calls give a partition of the reference genome $\mathcal{RG}$ into non-overlapping genomic intervals, each with a distinct copy number. We first remove centromeric intervals and select candidate seed intervals as the intervals a_i whose copy number is at least $\max(4.0 + \texttt{CN\_chr\_arm}(a_i), 6.0)$, where $\texttt{CN\_chr\_arm}(a_i)$ is the average (length-weighted) copy number of all intervals on the same chromosome arm as a_i. We merge adjacent candidate intervals and candidate intervals on the same chromosome and within $2 * 10^5$bp ($= 2\delta$, see below) distance. Among the merged intervals we finally select the ones with aggregated size at least 10^5bp ($= \delta$) as seed intervals. If there are no seed amplified intervals, CoRAL stops by reporting no focal amplifications in the input sample. Otherwise CoRAL proceeds with amplified interval search, breakpoint graph construction and cycle decomposition.

Amplified Interval Search. Let Δ be the maximum length of (amplified) genomic segment in an amplicon without breakpoints in the middle. According to TCGA and PCAWG data from (Kim et al. 2020), we set Δ to 10^6bp. Let δ be the size of flanking region surrounding an amplified interval. Following from (Deshpande et al. 2019) we set δ to 10^5bp. We present the pseudocode of amplified interval search, given a seed interval list $\mathcal{I}_s$, as follows.

Breakpoint Clustering. Define a "match" between two breakpoints $\text{bp}_1 = (s_1, p_1, o_1, s_2, p_2, o_2)$ and $\text{bp}_2 = (s'_1, p'_1, o'_1, s'_2, p'_2, o'_2)$ when they have the same chromosome, orientation, and close positions, i.e., $s_1 = s'_1$, $o_1 = o'_1$, $s_2 = s'_2$, $o_2 = o'_2$, $|p_1 - p'_1| \leq \texttt{bp\_distance\_cutoff}$ and $|p_2 - p'_2| \leq \texttt{bp\_distance\_cutoff}$, for some $\texttt{bp\_distance\_cutoff}$. We cluster a collection of breakpoints B through two steps. First we compute crude clusters with the following greedy strategy and a large $\texttt{bp_distance_cutoff} = 2000$bp (the largest possible distance given by the chimeric alignments between two reads which support a single breakpoint - see (Sedlazeck et al. 2018)): start with an empty set of clusters; for each breakpoint bp, if there exists a cluster $\mathcal{C}$ containing another breakpoint bp' which matches bp, then add the breakpoint bp to cluster $\mathcal{C}$; otherwise start a new cluster $\mathcal{C}'$ and add bp to $\mathcal{C}'$.

We then refine each crude cluster and compute the exact breakpoint. For each cluster $\mathcal{C}$ resulting from the above step, we compute the average μ_1 and standard deviation σ_1 of the first ("smaller") position p_1, as well as μ_2 and σ_2 of the second ("larger") position p_2. Then we remove the 'outlier' breakpoints in $\mathcal{C}$ with a smaller $\texttt{bp_distance_cutoff} = 100$bp, when $p_1 < \mu_1 - \max(3 * \sigma_1, \texttt{bp\_distance\_cutoff})$, or $p_1 > \mu_1 + \max(3 * \sigma_1, \texttt{bp\_distance\_cutoff})$, or $p_2 < \mu_2 - \max(3 * \sigma_2, \texttt{bp\_distance\_cutoff})$, or $p_2 > \mu_2 + \max(3 * \sigma_2, \texttt{bp\_distance\_cutoff})$. If the remaining cluster size is still at least $\texttt{bp\_clustersize\_cutoff}$, we keep the breakpoint corresponding to cluster $\mathcal{C}$, otherwise we split $\mathcal{C}$ into four subclusters: (i) breakpoints with $p_1 < \mu_1$ and $p_2 < \mu_2$; (ii) $p_1 < \mu_1$ and $p_2 > \mu_2$; (iii) $p_1 > \mu_1$ and $p_2 < \mu_2$; and (iv) $p_1 > \mu_1$ and $p_2 > \mu_2$. The subclusters with size at least $\texttt{bp\_clustersize\_cutoff}$ are repeated with the above procedure and otherwise are discarded. The final breakpoint positions for a cluster $\mathcal{C}$ is determined by the *mode* of p_1 and p_2 in $\mathcal{C}$. If there are multiple modes then we use the average positions. The $\texttt{bp\_clustersize\_cutoff}$ is determined by the maximum of 3 and haploid coverage to avoid false positive chimerisms in long read alignments. See below how CoRAL estimates the haploid coverage in a tumor sample.

Algorithm 1 AmplifiedIntervalSearch($\mathcal{I}_s, \delta, \Delta,$ `bp_clustersize_cutoff`) ▷ $\mathcal{I}_s$: seed intervals

```
for seed interval $a_i = (s_i, l_i, r_i) \in \mathcal{I}_s$ do
    $\mathcal{I}[a_i] \leftarrow -1$        ▷ $\mathcal{I}$: maps each amplified interval to an amplicon_id
end for
$\mathcal{E} \leftarrow$ empty map        ▷ $\mathcal{E}$: maps a pair of amplified intervals to a set of breakpoints
amplicon_id $\leftarrow 1$
for $a_i \in \mathcal{I}$ do
    if $\mathcal{I}[a_i] == -1$ then
        $L \leftarrow [a_i]$        ▷ $L$: priority queue used in current amplicon search
        while $L \neq \emptyset$ do
            $a_{\text{next}} = (s_{\text{next}}, l_{\text{next}}, r_{\text{next}}) \leftarrow L$.pop()
            if $\mathcal{I}[a_{\text{next}}] == -1$ then
                $\mathcal{I}[a_{\text{next}}] \leftarrow$ amplicon_id
            end if
            $B \leftarrow \{\text{bp} = (s_1, p_1, o_1, s_2, p_2, o_2) \mid$ either $p_1$ or $p_2$ overlaps with $a_{\text{next}}\}$
            Cluster breakpoints in $B$ with bp_clustersize_cutoff
            for breakpoint cluster $\text{bp}_{\mathcal{C}}$ from $B$ do
                $p \leftarrow$ the breakpoint position $p_1$ or $p_2$ from the cluster $\text{bp}_{\mathcal{C}}$ which does not overlap with $a_{\text{next}}$
                if $p$ exists then
                    if $p$ overlaps with some $a_j \in \mathcal{I}$ then
                        if $\mathcal{I}[a_j] == -1$ then
                            $L$.append($a_j$)
                        end if
                        $\mathcal{E}[(a_{\min(\text{next},j)}, a_{\max(\text{next},j)})] \leftarrow \mathcal{E}[(a_{\min(\text{next},j)}, a_{\max(\text{next},j)})] \cup \{\text{bp}_{\mathcal{C}}\}$
                    else
                        if $p$ is amplified then
                            $s_p \leftarrow$ start position of the CNV segment including $p$
                            $e_p \leftarrow$ end position of the CNV segment including $p$
                            $a_{\text{new}} \leftarrow (\text{chr}_p, \max(s_p - \delta, p - \Delta), \min(e_p + \delta, p + \Delta))$
                        else
                            $a_{\text{new}} \leftarrow (\text{chr}_p, p - \delta, p + \delta)$
                        end if
                        $L$.append($a_{\text{new}}$)
                        $\mathcal{I}[a_{\text{new}}] \leftarrow -1$
                        $\mathcal{E}[(a_{\text{next}}, a_{\text{new}})] \leftarrow \{\text{bp}_{\mathcal{C}}\}$
                    end if
                end if
            end for
        end while
        amplicon_id $\leftarrow$ amplicon_id $+ 1$
    end if
end for
```

S3. Copy Number Assignment

Estimating diploid coverage. The CN assignment of CoRAL requires an estimation of diploid coverage θ_{LR}. The estimation can be derived from CNV calls used for detecting seed amplified intervals, with the assumption that majority of the donor genome is not amplified. Recall that CNV calls give a partition of the reference genome $\mathcal{RG}$ into non-overlapping genomic intervals, each with a distinct copy number. To estimate θ_{LR} CoRAL first sorts these intervals according to their predicted copy numbers, and locate the interval $a_i = (s_i, l_i, r_i)$ at the 40-th percentile in the sorted list. If the length of a_i is less than 10^7bp, CoRAL iteratively includes the interval a_{i-1} and a_{i+1} in the sorted list, until the aggregate size of the included intervals is at least 10^7bp. CoRAL computes θ_{LR} as the (length-weighted) average long read coverage of all selected intervals centered at a_i.

Maximum likelihood CN assignment. Given θ_{LR}, we model the total number of nucleotides N_e on each sequence edge $e \in E_s$ as a normal distribution with mean and variance both $\theta_{\mathrm{LR}} \cdot \mathrm{CN}(e) \cdot l(e)$, where $l(e)$ denotes the length (in bp) of the sequence edge

$$P(N_e \mid \mathrm{CN}(e)) = \mathcal{N}(\mu = \theta_{\mathrm{LR}} \cdot \mathrm{CN}(e) \cdot l(e), \sigma^2 = \theta_{\mathrm{LR}} \cdot \mathrm{CN}(e) \cdot l(e)) \ , e \in E_s; \tag{S3.1}$$

and the number of reads n_e^{LR} supporting each concordant and discordant edge $e \in E_c \cup E_d$ as a Poisson (similar to (Medvedev et al. 2010; Deshpande et al. 2019)) with mean $\theta_{\mathrm{LR}} \cdot \mathrm{CN}(e)$

$$P(n_e^{\mathrm{LR}} \mid \mathrm{CN}(e)) = \frac{e^{-\theta_{\mathrm{LR}} \cdot \mathrm{CN}(e)} (\theta_{\mathrm{LR}} \cdot \mathrm{CN}(e))^{n_e^{\mathrm{LR}}}}{n_e^{\mathrm{LR}}!}, \ e \in E_c \cup E_d. \tag{S3.2}$$

To estimate CN, CoRAL computes the maximum likelihood $\mathcal{L}$ of CN using the joint distribution of observed number of nucleotides on each sequence edge and the observed read counts on each concordant/discordant edge

$$\mathcal{L}(\mathrm{CN}) = \prod_{e \in E_s} P(N_e \mid \mathrm{CN}(e)) \cdot \prod_{e \in E_c \cup E_d} P(n_e^{\mathrm{LR}} \mid \mathrm{CN}(e)), \tag{S3.3}$$

with the constraint that CN is balanced for each node v (by rewriting equation S1.1), i.e.,

$$\sum_{e \in E_s(v)} \mathrm{CN}(e) = \sum_{e \in E_c \cup E_d(v)} \mathrm{CN}(e), \ \forall v \neq s, t \in V, \tag{S3.4}$$

where $E_s(v)$, $E_c(v)$, and $E_d(v)$ stand for the sequence edge, concordant edge, and discordant edges connected to node v, respectively. Edges connected to the source nodes s and t do not contribute to the likelihood (objective) function, nor to the constraints. The (convex) optimization problem was solved using CVXOPT package (`https://github.com/cvxopt/cvxopt`).

S4. Cycle Decomposition

In cycle decomposition we decompose an amplicon $\mathcal{G}$ into a collection of cycles and s, t-walks, with high copy numbers. For all sequence edges $(u, v) \in E_s$, define the *length-weighted-copy-number* using $\mathrm{C}_l(u, v) = \mathrm{CN}(u, v) \cdot l(u, v)$, where $l(u, v)$ denotes the length (in bp) of the corresponding segment. Similarly, for graph $\mathcal{G}$.

$$\mathrm{C}_l(\mathcal{G}) = \sum_{(u,v) \in E_s} \mathrm{C}_l(u, v) \tag{S4.1}$$

The MIQCP for cycle extraction works with 3 parameters: k as the maximum number of walks; α as the minimum fraction of length-weighted copy number explained, and β as the minimum fraction of path constraints satisfied. Of these, k is learned starting with $k = 1$, according to two modes. In the **FullQP** mode, the MIQCP attempts a solution with at most k walks that satisfy other constraints, or returns 'infeasible.' The value of k is doubled until feasibility is reached or $k > |E|$. The **greedy mode** is described below. We implement both quadratic programs with through the python3 interface of Gurobi 10.0.1.

We use the following **key** variables.

- $w_i \in \mathbb{Q} \geq 0$: denotes the copy number for walk W_i ($1 \leq i \leq k$); an auxiliary variable $z_i \in \{0, 1\}$ indicates if $w_i > 0$;
- $x_{uvi} \in \mathbb{Z} \geq 0$ represents the number of times walk W_i traverses (u, v) for each edge $(u, v) \in E$ and $1 \leq i \leq k$;
- $P_j \in \{0, 1\}$ indicates if subwalk constraint p_j is satisfied for $1 \leq j \leq m$;

The MIQIP(k, α, β) objective is given by:

$$\mathbf{min} \sum_{i=1}^{k} z_i - \frac{1}{\mathrm{C}_l(\mathcal{G})} \sum_{i=1}^{k} \sum_{(u,v) \in W_i \cap E_s} w_i \cdot x_{uvi} \cdot l(u, v) - \frac{1}{m} \sum_{j=1}^{m} P_j \tag{S4.2}$$

subject to the constraints:

$$w_i \leq z_i \cdot \max_{(u,v) \in E} \mathrm{CN}(u, v) \tag{S4.3}$$

$$\sum_{i=1}^{k} \sum_{(u,v) \in W_i \cap E_s} w_i \cdot x_{uvi} \cdot l(u, v) \geq \alpha \cdot \mathrm{C}_l(\mathcal{G}) \tag{S4.4}$$

$$\sum_{j=1}^{m} P_j \geq \beta \cdot m \tag{S4.5}$$

In addition, the MIQIP satisfies a number of auxiliary constraints that constrain the cycles and walks to satisfy normal definitions, and those require the following auxiliary variables:

- $c_{vi} \in \{0, 1\}$ for node $v \in V - \{s, t\}$ and $1 \leq i \leq k$. $c_{v,i} = 1$ iff the walk W_i forms a cycle starts (and ends) with node v, if W_i exists;
- $p_{ij} \in \{0, 1\}$, for $1 \leq i \leq k, 1 \leq j \leq m$ indicates if subwalk constraint p_j is satisfied by walk W_i;
- $0 \leq d_{vi} \leq |V|$ for each node $v \in V, 1 \leq i \leq k$, starting with a number 1 for the initial node and incrementing for the next node in the cycle/walk. It is used to ensure connectivity;
- $y^{+}_{uvi} \in \{0, 1\}$, $y^{-}_{uvi} \in \{0, 1\}$ for each edge $(u, v) \in E$ and $1 \leq i \leq k$, is also used to ensure connectivity.

Additional Constraints.

1. Each W_i should form a valid walk of alternating sequence and breakpoint edges. In other words, for each node $v \in V - \{s, t\}$, the sum of x_{uvi} from sequence edges $(u, v) \in E_s$ it connects to should equal to the sum of x_{uvi} from breakpoint edges $(v, w) \in E_c \cup E_d$ it connects to.

$$\sum_{(u,v) \in E_s} x_{uvi} - \sum_{(v,w) \in E_c \cup E_d} x_{vwi} = 0, \ \forall v \in V - \{s, t\}, \ \forall i = 1, \cdots, k \tag{S4.6}$$

2. The total CN of all cycles/walks passing through an edge $(u, v) \in E$ is at most $\mathrm{CN}(u, v)$.

$$\sum_i w_i \cdot x_{uvi} \leq \mathrm{CN}(u, v),\ \forall (u, v) \in E \tag{S4.7}$$

3. We require that each cycle/walk traverses through a discordant edge $(u, v) \in E_d$ at most $R(u, v)$ times, otherwise cycles were not possible.

$$x_{uvi} \leq R(u, v),\ \forall (u, v) \in E_d \tag{S4.8}$$

$R(u, v)$ can be a small, fixed number for all $(u, v) \in E_d$, e.g., 2 to allow a discordant edge to be traversed in each direction as the graph is undirected. However, by default CoRAL computes an empirical $R(u, v)$ for each $(u, v) \in E_d$ by clustering the number of reads $n^{\mathrm{LR}}_{(u,v)}$ supporting the discordant edge with the constraint that two observations $n^{\mathrm{LR}}_{e_1}, n^{\mathrm{LR}}_{e_2}$ must belong to different clusters if one of them is at leats 5 times larger than the other, i.e., $n^{\mathrm{LR}}_{e_1} \geq 5 \cdot n^{\mathrm{LR}}_{e_2}$ or $n^{\mathrm{LR}}_{e_2} \geq 5 \cdot n^{\mathrm{LR}}_{e_1}$. In case there exists some edges with $R(u, v) > 1$, the constraint S4.11 below prevents every edge in a cycle being repeated multiple times.

4. Each walk W_i either forms a cycle starting at node v, or starts at s and ends at t if it exists. If W_i forms a cycle we require that there exists one concordant or discordant edge connected to c_{vi} which occurs only once in the cycle.

$$\sum_{(s,v)\in E} x_{svi} + \sum_v c_{vi} \leq 1 \quad \forall i = 1, \cdots, k \tag{S4.9}$$

$$\sum_{(s,u)\in E} x_{sui} = \sum_{(v,t)\in E} x_{vti} \quad \forall i = 1, \cdots, k \tag{S4.10}$$

$$\sum_{(u,v)\in E_c\cup E_d} x_{uvi} \cdot c_{vi} \leq 1 \quad \forall v \in V \text{ and } i = 1, \cdots, k \tag{S4.11}$$

5. x_{uvi} and z_i are consistent. In other words $z_i = 1$ if and only if there exists some $x_{uvi} > 0$. Since x_{uvi} are not binary, the consistency between x_{uvi} and z_i can be guaranteed through y^+_{uvi} or y^-_{uvi}.

$$z_i \geq y^+_{uvi}\ \forall (u, v) \in E \text{ and } i = 1, \cdots, k \tag{S4.12}$$

$$z_i \geq y^-_{uvi}\ \forall (u, v) \in E \text{ and } i = 1, \cdots, k \tag{S4.13}$$

$$y^+_{uvi} + y^-_{uvi} \leq x_{uvi}\ \forall (u, v) \in E \text{ and } i = 1, \cdots, k \tag{S4.14}$$

6. Connectivity. The idea is to use d_{vi} to encode the "discovery order" of the nodes in walk W_i. If W_i is a cycle then we start with the node v where $c_{vi} = 1$; otherwise we start with the source node s. d_{vi} for the starting node v is set to 1. Each node v (except the starting node) in W_i is assumed to be "discovered" **uniquely** by another already "discovered" node u through edge (u, v), satisfying $d_{vi} \geq d_{ui} + 1$. As breakpoint graph is undirected, we assume an order $<$ of nodes and for each edge (u, v) we introduce two binary variables y^+_{uvi} or y^-_{uvi} indicating respectively the larger node in u, v is discovered from the smaller node through edge (u, v); or the smaller node is discovered from the larger node through edge (u, v). Specifically, if v is discovered from u and $v > u$ or u is discovered from v and $v < u$ then $y^+_{uvi} = 1$; if v is discovered from u and $v < u$ or u is discovered from v and $v > u$ then $y^-_{uvi} = 1$; in all other cases $y^+_{uvi} = y^-_{uvi} = 0$. The nodes do not belong to W_i also have $d_{vi} = 0$ and $y^+_{uvi} = y^-_{uvi} = 0$. We first set up the constraint for the starting node.

$$d_{vi} \geq c_{vi},\ \forall v \in V,\ \text{and } i = 1, \cdots, k \tag{S4.15}$$

$$d_{si} + \sum_{v \in V} c_{vi} \leq 1, \ \forall i = 1, \cdots, k \tag{S4.16}$$

We require that $d_{vi} = 0$ if node v is not a part of W_i.

$$d_{vi} \leq |V| \cdot \sum_{(u,v) \in E} x_{uvi}, \ \forall v \in V, \text{ and } i = 1, \cdots, k \tag{S4.17}$$

Fold-back edges (self-loops) can not have positive y^+_{uvi} or y^-_{uvi}.

$$y^+_{uui} = 0, \ \forall (u,u) \in E_d \text{ and } i = 1, \cdots, k \tag{S4.18}$$

$$y^-_{uui} = 0, \ \forall (u,u) \in E_d \text{ and } i = 1, \cdots, k \tag{S4.19}$$

Each node can be discovered from at most one neighboring node.

$$\sum_{(u,v) \in E, u<v} y^+_{uvi} + \sum_{(u,v) \in E, u>v} y^-_{uvi} \leq 1, \ \forall v \in V \text{ and } i = 1, \cdots, k \tag{S4.20}$$

If a node v is included in walk W_i and it is not the starting node of a cycle, then it must be discovered through some y^+_{uvi} or y^-_{vui}.

$$\sum_{(u,v) \in E} x_{uvi} \geq 1 \wedge c_{vi} = 0 \rightarrow \sum_{(u,v) \in E, u<v} y^+_{uvi} + \sum_{(u,v) \in E, u>v} y^-_{uvi} \geq 1, \ \forall v \in V \text{ and } i = 1, \cdots, k \tag{S4.21}$$

Equation (S4.21) can be rewritten as a quadratic constraint, as follows. For all $v \in V$ and $i = 1, \cdots, k$,

$$\left(\sum_{(u,v) \in E, u<v} y^+_{uvi} + \sum_{(u,v) \in E, u>v} y^-_{uvi} \right) \cdot C|E| + \sum_{(u,v) \in E} x_{uvi} \cdot c_{vi} \geq \sum_{(u,v) \in E} x_{uvi}, \tag{S4.22}$$

where C is a large constant representing the maximum number of times that any edge $(u,v) \in E$ can be traversed by a cycle or walk (e.g., C can be set to max degree of $\mathcal{G} \cdot \max_{(u,v) \in E_d} R(u,v)$). Finally, we connect y^+_{uvi} and y^-_{uvi} with d_{vi}. If a node v is included in walk W_i and it is not the starting node of a cycle, then $d_{vi} \geq d_{ui} + 1$ for the node u it was discovered from. For all $v \in V$ and $i = 1, \cdots, k$

$$\begin{aligned} \left(\sum_{(u,v) \in E, u<v} y^+_{uvi} \cdot (d_{vi} - d_{ui}) + \sum_{(u,v) \in E, u>v} y^-_{uvi} \cdot (d_{vi} - d_{ui}) \right) \cdot C|E| + \\ \sum_{(u,v) \in E} x_{uvi} \cdot c_{vi} \geq \sum_{(u,v) \in E} x_{uvi}. \end{aligned} \tag{S4.23}$$

7. **Subwalk constraints.** We enforce a weak constraint by requiring each walk $p_j \in \mathcal{P}$ as a subgraph of the graph induced by some walk W_i.

$$P_j \geq p_{ij}, \ \forall i = 1, \cdots, k \text{ and } j = 1, \cdots, m \tag{S4.24}$$

$$x_{uvi} \geq p_{ij} \cdot p_j(u,v), \ \forall (u,v) \in E \cap p_j \text{ and } i = 1, \cdots, k \text{ and } j = 1, \cdots, m \tag{S4.25}$$

where $p_j(u,v)$ the number of times walk p_j passes through an edge (u,v).

Subwalk constraints from long reads. To compute subwalk constraints we extract all long reads mapped within the amplified intervals defined by the breakpoint graph $\mathcal{G}$, and map each of them to $\mathcal{G}$. We filter out reads which could not be fully mapped to the breakpoint graph, due to additional breakpoints or partially non-overlap with any sequence edge. Each of the remaining reads mapped to $\mathcal{G}$ should give a walk in $\mathcal{G}$ starting and ending with sequence edges. We further filter out walks where the first or last sequence edge overlaps with the corresponding read by less than 500bp; and then walks with at most 3 edges (which only cover one breakpoint). Finally, we filter out walks that form a subwalk of any other walk and return the remaining walks as the subwalk constraints $\mathcal{P}$ to be used in cycle extraction, either full QP or greedy QP.

MIQIP-greedy. Let $\bar{\mathcal{P}} = \{j \mid \text{path } p_j \text{ is not satisfied by any previously selected walk}\}$. The full greedy MIQCP to identify the next walk W_i is given by:

$$\mathbf{max} \quad \sum_{(u,v)\in W_i \cap E_s} w \cdot x_{uv} \cdot l(u,v) + \gamma \sum_{j \in \bar{\mathcal{P}}} P_j \tag{S4.26}$$

subject to

$$w \leq \max_{e\in E}\{\mathrm{C}_l(e)\} \cdot z \tag{S4.27}$$

$$\sum_{(u,v)\in E_s} x_{uv} - \sum_{(v,w)\in E_c \cup E_d} x_{vw} = 0,\ \forall v \in V - \{s,t\} \tag{S4.28}$$

$$x_{uv} \cdot w \leq \text{remaining CN}(u,v),\ \forall (u,v) \in E \tag{S4.29}$$

$$x_{uv} \leq R(u,v),\ \forall (u,v) \in E_d \tag{S4.30}$$

$$\sum_{(s,v)\in E} x_{sv} + \sum_{v} c_v \leq 1 \tag{S4.31}$$

$$\sum_{(s,u)\in E} x_{su} = \sum_{(v,t)\in E} x_{vt} \tag{S4.32}$$

$$\sum_{(u,v)\in E_s} x_{uv} \cdot c_v \leq 1,\ \forall v \in V \tag{S4.33}$$

$$z \geq y^+_{uv},\ \forall (u,v) \in E \tag{S4.34}$$

$$z \geq y^-_{uv},\ \forall (u,v) \in E \tag{S4.35}$$

$$y^+_{uv} + y^-_{uv} \leq x_{uv},\ \forall (u,v) \in E \tag{S4.36}$$

$$d_v \geq c_v,\ \forall v \in V \tag{S4.37}$$

$$d_s + \sum_{v\in V} c_v \leq 1 \tag{S4.38}$$

$$d_v \leq |V| \cdot \sum_{(u,v)\in E} x_{uv},\ \forall v \in V \tag{S4.39}$$

$$y^+_{uu} = 0,\ \forall (u,u) \in E_d \tag{S4.40}$$

$$y^-_{uu} = 0,\ \forall (u,u) \in E_d \tag{S4.41}$$

$$\sum_{(u,v)\in E, u<v} y^+_{uv} + \sum_{(u,v)\in E, u>v} y^-_{uv} \leq 1,\ \forall v \in V \tag{S4.42}$$

$$\left(\sum_{(u,v)\in E, u<v} y^+_{uv} + \sum_{(u,v)\in E, u>v} y^-_{uv}\right)\cdot C|E| + \sum_{(u,v)\in E} x_{uv}\cdot c_v \geq \sum_{(u,v)\in E} x_{uv},\ \forall v\in V \tag{S4.43}$$

$$\left(\sum_{(u,v)\in E, u<v} y^+_{uv}\cdot(d_v-d_u) + \sum_{(u,v)\in E, u>v} y^-_{uv}\cdot(d_v-d_u)\right)\cdot C|E| + \sum_{(u,v)\in E} x_{uv}\cdot c_v \geq \sum_{(u,v)\in E} x_{uv},\ \forall v\in V \tag{S4.44}$$

$$x_{uv} \geq P_j\cdot p_j(u,v),\ \forall (u,v)\in E\cap p_j \text{ and } j=1,\cdots,m \tag{S4.45}$$

$$z, c_v, y^+_{uv}, y^-_{uv}, P_j \in \{0,1\}; x_{uv}, d_v\in\mathbb{Z}; 0\leq x_{uv}\leq C; 0\leq d_v\leq |V|; w\geq 0 \tag{S4.46}$$

S5. Simulating amplicon structures with ecSimulator

We utilized an updated version of ecSimulator (Luebeck et al. 2020) (version 0.6.0, `https://github.com/AmpliconSuite/ecSimulator`) to simulate ecDNA genome structures derived from three different contexts and simulated both long and short reads from those structures at varying copy number levels.

In brief, ecSimulator uses a user-specified YAML input to set simulation parameters, allowing the user to specify properties such as the number of genomic intervals in the simulated ecDNA, the number of possible locations of breakpoints, and the rates of SV types (deletion, duplication, inversion, translocation, foldback). We utilized ecSimulator's default parameters for SV type frequency. ecSimulator supports the simulation of ecDNA derived from three different modes of genesis. First, the episome model, whereby the structure is initialized with only head-to-tail closure of the interval(s). Second, the two-foldback model where the interval is bound on left and right by a foldback SV (inverted duplication). Third, a chromothriptic model, whereby intervals are separated by a deletions and then closed head-to-tail, simulating the oscillating CN states observed in chromothripsis. To simulate the internal rearrangements of the genome structure observed on ecDNA intervals, ecSimulator first assigns n breakpoints randomly throughout the intervals according to the number of possible breakpoints desired by the user. By pre-assigning possible breakpoint locations, it is possible to easily create structures containing multiple copies of a breakpoint or to enable breakpoint re-use. ecSimulator then performs multiple rounds of SV boundary assignment and rearrangement, with the default number of rounds being $\frac{n}{2}$. A random number of consecutive sub-segments of the intervals are selected, where sub-segments are defined as pieces of the intervals separated by the pre-assigned breakpoint locations. The selected sub-segments then undergo random assignment of the SV type being applied, and the assigned SV type is simulated inside the structure on those selected segments.

To simulate reads we maintained the following default parameters:

target_size: 2,000,000
mean_segment_size: 150,000
num_intervals: "auto"
same_chromosome: False (allowing segments to be recombined across chromosomes)
allow_interval_reuse: True (allowing higher multiplicity in an amplicon)
viral_insertion: False
del: 0.6 (probability of deletion)
dup: 0.5 (probability of duplication)
inv: 0.4 (probability of inversion)
trans: 0.4 (probability of translocation)
fback: 0.05 (probability of an inverted duplication)

In addition to these default parameters, we simulated amplicons from the chromothripsis, episomal, or two-foldback origins. For each origin, we simulated 5 replicates of an amplicon with 1, 3, 5, 10, or 20 breakpoints resulting in a dataset of 75 simulated amplicons.

S6. Whole Genome Sequencing (WGS)

Public data sources. We obtained sequencing data from the following public repositories. Illumina data for COLO320-DM (Wu 2019) was obtained from SRX5055021; Illumina data for COLO320-HSR (Wu 2019) was obtained from SRX5930165. Illumina data for GBM39 (Wu 2019) was obtained from SRX5055022; Illumina data for GBM39-HSR (Wu 2019) was obtained from SRX5930166; Illumina data for CHP-212 was obtained from SRX8044100; Illumina data for COLO320-DM (Hung 2021) was obtained from SRX11096731. Long-read Nanopore data for COLO320-DM (Hung 2021) was obtained from SRX9346575; Long-read Nanopore data for CHP-212 was obtained from SRX8044102.

We additionally sequenced the other 4 Illumina samples (COLO320-DM (mono), GBM39 (mono), PC3-DM (mono), PC3-HSR (mono)) and 8 Nanopore samples (COLO320-DM (Wu 2019), COLO320-HSR (Wu 2019), GBM39 (Wu 2019), GBM39-HSR (Wu 2019), COLO320-DM (mono), GBM39 (mono), PC3-DM (mono), PC3-HSR (mono)), as described in the following sections (see "Short-read WGS" and "Nanopore Long-read WGS").

Deriving monoclonal cell lines. The following approaches were taken to derive stable monoclonal cell lines for this study:

1. **PC3:** PC3 cell line with a mixed pooled of cells containing *MYC* amplification as either ecDNA (for PC3-DM) or HSR (for PC3-HSR) were seeded onto a 96-well plate at a density of 0.5 cell/well, in an attempt to have a maximum of one cell seeded per well. Cells were then expanded and iteratively expanded into a 24-well plate, 6-well plate, then 10 cm dish over the course of one month. PC3 cells were maintained in Dulbecco's modified Eagle's medium (DMEM; Corning, #10-013-CV) supplemented with 10% fetal bovine serum (FBS; Hyclone, SH30396.03) and 1% pen-strep (PS; Thermo Fisher Scientific, 15140-122).
2. **GBM39:** GBM39 cell line with a mixed pooled of cells containing *EGFR*vIII amplification as ecDNA were sent to Cell Microsystems for monoclonal expansion. In brief, cells were subjected to TrypLE digestion to yield single cells, and each single cell was seeded onto a CellRaft Array using the CellRaft AIR® System. The cells were maintained and expanded as a monoclonal line. Neurosphere culture medium Dulbecco's modified Eagle's medium/nutrient mixture F-12 (DMEM/F12 1:1; Gibco, 11320-082) with 1% PS, GlutaMAX (Gibco, 35050061), B27 supplement (Gibco, 17504044), 20 ng/ml epidermal growth factor (EGF; Sigma-Aldrich, E9644), 20 ng/ml fibroblast growth factor (FGF; Peprotech) and 5 μg/ml (Sigma-Aldrich, H3149-500KU) was used throughout to maintain the culture.
3. **COLO320-DM:** A recently monoclonalized cell line of COLO320-DM was received as a gift from the Mischel group.

Short-read WGS. WGS libraries were prepared by DNA tagmentation. We first transposed it with Tn5 transposase produced as previously described, in a 50-µl reaction with TD buffer, 10ng DNA and 1 µl transposase. The reaction was performed at 50°C for 5 minutes, and transposed DNA was purified using Zymo DNA Clean & Concentrate kit (Zymo, 1159U33). Libraries were generated by 7 rounds of PCR amplification using NEBNext High-Fidelity 2× PCR Master Mix (NEB, M0541L), purified using SPRIselect reagent kit (Beckman 635 Coulter, B23317) with double

size selection (0.8× right, 1.2× left) and sequenced on the Illumina Nextseq 550 platform. Reads were trimmed of adapter content with Trimmomatic (Bolger et al. 2014) (version 0.39), aligned to the hg38 genome using BWA MEM (Li and Durbin 2009) (version 0.7.17-r1188), and PCR duplicates removed using Picard's MarkDuplicates (version 2.25.3).

Nanopore Long-read WGS. We performed default long-read sequencing on COLO320-HSR, GBM39 (mono), and PC3-DM (mono). To do so, high molecular weight (HMW) genomic DNA from approximately 2 million cells was extracted using the Qiagen Puregene DNA Kit (Qiagen 158023) and prepared for long-read sequencing using the Oxford Nanopore Ligation Sequencing Kit V14 (Oxford Nanopore Technologies SQK-LSK114) according to the manufacturer's instructions.

We performed Ultralong seqeuncing on COLO320-DM (mono) and PC3-HSR (mono). To do so, HMW genomic DNA was extracted from approximately 6 million cells using the NEB HMW DNA Extraction Kit for Cells & Blood (NEB T3050) and prepared for sequencing using the Oxford Nanopore Ultra-Long DNA Sequencing Kit V14 (Oxford Nanopore Technologies SQK-ULK114) according to the manufacturer's instructions. Libararies were sequenced on a PromethION (Oxford Nanopore Technologies) using a 10.4.1 flow cell (Oxford Nanopore Technologies FLO-PRO114M). Basecalling from raw POD5 files was performed using Dorado (Oxford Nanopore Technologies, version 0.2.1) and aligned to GRCh38 using minimap2 (Li 2018).

In addition, we performed long-read sequencing on non-monoclonalized GBM39 and GBM39-HSR as follows: HMW genomic DNA was extracted using a MagAttract HMW DNA Kit (Qiagen 67563) and prepared for long-read sequencing using a Ligation Sequencing Kit (Oxford Nanopore Technologies SQK-LSK109) according to the manufacturer's instructions. Libraries were sequenced on a PromethION (Oxford Nanopore Technologies) using a 9.4.1 flow cell (Oxford Nanopore Technologies FLO-PRO002). Bases were called from FAST5 files using Guppy (Oxford Nanopore Technologies, v.2.3.7) and read were aligned to GRCh38 using minimap2 (Li 2018).

S7. Amplicon comparison statistics

Breakpoint-graph accuracy. We evaluate the proportion of breakpoint edges captured for a reconstruction as follows: let E_d^r be the set of discordant edges in a reconstructed breakpoint graph $\mathcal{G}_r$ and E_d^t be the set of discordant edges in a simulated breakpoint graph $\mathcal{G}_t$. For each edge $(s_1^t, p_1^t, o_1^t, s_2^t, p_2^t, o_2^t) \in E_d^t$ we evaluate if a similar edge appears in the set of reconstructed edges E_d^r. Specifically, an an edge is deemed similar if the orientations are identical and the positions differ by at most 100bp - i.e., $|p_1^r - p_1^t| \leq 100$ and $|p_2^r - p_2^t| \leq 100$, where p_*^t is the true coordinate and p_*^r is the reconstructed coordinate. The proportion of true edges recovered is reported.

Cycle-interval overlap. Let $\mathcal{I}^t = \{(s_i^t, l_i^t, r_i^t)\}$ be the set of intervals covered by the simulated cycle and let $\mathcal{I}^{r,n} = \{(s_i^{r,k}, l_i^{r,k}, r_i^{r,k})\}$ be the set of intervals covered by the n-th cycle returned by a reconstruction algorithm. (Recall that CoRAL and AA return a set of possible cycles). Let $\delta(\mathcal{I}_i, \mathcal{I}_j)$ be a function that returns the length (in nucleotides) of the overlapping region between two intervals $\mathcal{I}_i$ and $\mathcal{I}_j$ (if $s_i \neq s_j$ then $\delta(\mathcal{I}_i, \mathcal{I}_j) = 0$); let $\ell(\mathcal{I}_j)$ indicate the length (in nucleotides) of the interval $\mathcal{I}_j$ (i.e., $|r_i - l_i|$). Then the Cycle-interval overlap is for the n-th cycle is defined as

$$O_n = \frac{\sum_{j \in |\mathcal{I}^t|} \sum_{k \in |\mathcal{I}^{r,n}|} \delta(\mathcal{I}_j^t, \mathcal{I}_k^{r,n})}{\sum_{j \in |\mathcal{I}^t|} \ell(\mathcal{I}_j^t) + \sum_{k \in |\mathcal{I}^{r,n}|} \ell(\mathcal{I}_k^{r,n})}$$

For a given simulated amplicon, we report best overlap statistic found across all returned reconstructions.

Cyclic Longest Common Subsequence (LCS). Let W_t be a true, simulated cycle defined by an ordered sequence of intervals $\mathcal{I}^t = \{(s_i^t, l_i^t, r_i^t)\}$, and let W_r^n be the n-th cycle defined by the ordered sequence of intervals $\mathcal{I}^{r,n} = \{(s_i^r, l_i^r, r_i^r)\}$ returned by a cycle decomposition algorithm (as above, recall that CoRAL and AA return a set of possible cycles). Let $\text{LCS}(\mathcal{I}^t, \mathcal{I}^{r,n}(j))$ be defined as the longest common subsequence between the two ordered interval sets (in nucleotides). Note that the common subsequence does not need to be contiguous. For example, for the sequence $\mathcal{I}_1 = (1, 2, 3, 4, 5)$ and $\mathcal{I}_2 = (1, 3, 4, 5, 6)$, the LCS would be $(1, 3, 4, 5)$.

To account for cycle rotation, we also consider the rotated cycle $W(j)$ containing m intervals where the interval set is rotated around the index j: $\mathcal{I} = \{\mathcal{I}_j, \mathcal{I}_{j+1} \ldots \mathcal{I}_m, \mathcal{I}_1, \ldots \mathcal{I}_{j-1}\}$. In addition, we consider the reverse cycle whereby $\bar{W}$ consists of the reverse of all intervals: $\bar{\mathcal{I}} = \{\bar{\mathcal{I}}_m, \bar{\mathcal{I}}_{m-1}, \ldots, \bar{\mathcal{I}}_1\}$ and $\bar{\mathcal{I}}_i = (s_i, r_i, l_i)$.

Then, the cyclic longest common subsequence for a reconstructed cycle consisting of m intervals is defined as (only considering intervals that overlap between the true and reconstructed cycle):

$$\text{C-LCS}(W_t, W_r^n) = \frac{\max\left(\max_{j \in m} \text{LCS}(\mathcal{I}^t, \mathcal{I}^{r,n}(j)), \max_{j \in m} \text{LCS}(\mathcal{I}^t, \bar{\mathcal{I}}^{r,n}(j))\right)}{\sum_{k \in |\mathcal{I}^t|} \ell(\mathcal{I}_k^t)}$$

In words, this measure reports the longest common subsequence that can be found between the two cycles W_t and W_r^n while considering all possible rotations and reversals, and normalized to the length of the true cycle (in nucleotides).

Reconstruction Length Error. This statistic measures the differences in reconstructed cycle length and true cycle length. As before, let W_t be a true, simulated cycle defined by an ordered sequence of intervals $\mathcal{I}^t = \{(s_i^t, l_i^t, r_i^t)\}$, and let W_r^n be the n-th cycle defined by the ordered sequence of intervals $\mathcal{I}^{r,n} = \{(s_i^r, l_i^r, r_i^r)\}$. Furthermore, let L_* be the length of cycle W_* (where $*$ indicates t or some reconstruction W_{r^n})): $L_* = \sum_{k \in |\mathcal{I}^*|} \ell(\mathcal{I}_k^*)$

Then, the reconstruction length error for a particuler reconstruction W_r^n is defined as

$$R_{r^n} = \frac{(L_{r^n} - L_t)}{L_t}$$

The final value R_r is defined as the value for the best reconstruction: $R_r = \min_n R_{r^n}$. For clarity of presentation, we report $\log_2(1 + R_r)$.

$K-$**Heaviest Cycle Weight Ratio.** This statistic is agnostic to the true cycle and reflects the "entropy" of a given reconstruction, by measuring the fraction of total copy-number can be accounted for by the k heaviest cycles. Here, the length-weighted copy-number of the entire breakpoint graph (denoted as $\text{C}_l(\mathcal{G})$) is calculated from the sequence edges E_s reported in a breakpoint graph $\mathcal{G}$ and is defined in Eqn.(2) of the main text. The length-weighted copy-number of a particular cycle is defined as the sum of the length-weighted copy-number the intervals reported in a particular reconstructed cycle W_r^n that is defined by m total intervals contained in the set $\mathcal{I}^{r,n}$. Using similar notation as above, if $\text{C}_l(u, v)$ denotes the length-weighted copy-number of a particular interval covering (u, v), then we define the copy-number ratio of the n-th reconstructed cycle as:

$$\text{C}_l(W_r^n) = \frac{\sum_{i \in |\mathcal{I}^{r,n}|} \text{C}_l(l_i, r_i)}{\text{C}_l(\mathcal{G})}$$

Then, rank-ordering the N cycles returned by a cycle decomposition algorithm in descending order, we define the $k-$heaviest copy-number ratio as the sum of the k largest cycles (where W_r^i represents the i-th largest cycle):

$$C_l^{1\cdots k} = \sum_{i\in 1\cdots k} C_l(W_r^i)$$

References

Sergey Aganezov and Benjamin J Raphael. Reconstruction of clone-and haplotype-specific cancer genome karyotypes from bulk tumor samples. *Genome research*, 30(9):1274–1290, 2020.

Anthony M Bolger, Marc Lohse, and Bjoern Usadel. Trimmomatic: a flexible trimmer for illumina sequence data. *Bioinformatics*, 30(15):2114–2120, 2014.

Viraj Deshpande, Jens Luebeck, Nam-Phuong D Nguyen, Mehrdad Bakhtiari, Kristen M Turner, Richard Schwab, Hannah Carter, Paul S Mischel, and Vineet Bafna. Exploring the landscape of focal amplifications in cancer using AmpliconArchitect. *Nature communications*, 10(1):1–14, 2019.

Kevin Hadi, Xiaotong Yao, Julie M Behr, Aditya Deshpande, Charalampos Xanthopoulakis, Huasong Tian, Sarah Kudman, Joel Rosiene, Madison Darmofal, Joseph DeRose, et al. Distinct classes of complex structural variation uncovered across thousands of cancer genome graphs. *Cell*, 183(1): 197–210, 2020.

Hoon Kim, Nam-Phuong Nguyen, Kristen Turner, Sihan Wu, Amit D Gujar, Jens Luebeck, Jihe Liu, Viraj Deshpande, Utkrisht Rajkumar, Sandeep Namburi, et al. Extrachromosomal DNA is associated with oncogene amplification and poor outcome across multiple cancers. *Nature genetics*, 52(9):891–897, 2020.

Heng Li. Minimap2: pairwise alignment for nucleotide sequences. *Bioinformatics*, 34(18):3094–3100, 2018.

Heng Li and Richard Durbin. Fast and accurate short read alignment with Burrows–Wheeler transform. *bioinformatics*, 25(14):1754–1760, 2009.

Jens Luebeck, Ceyda Coruh, Siavash R Dehkordi, Joshua T Lange, Kristen M Turner, Viraj Deshpande, Dave A Pai, Chao Zhang, Utkrisht Rajkumar, Julie A Law, et al. AmpliconReconstructor integrates NGS and optical mapping to resolve the complex structures of focal amplifications. *Nature communications*, 11(1):4374, 2020.

Paul Medvedev, Marc Fiume, Misko Dzamba, Tim Smith, and Michael Brudno. Detecting copy number variation with mated short reads. *Genome research*, 20(11):1613–1622, 2010.

Fritz J Sedlazeck, Philipp Rescheneder, Moritz Smolka, Han Fang, Maria Nattestad, Arndt Von Haeseler, and Michael C Schatz. Accurate detection of complex structural variations using single-molecule sequencing. *Nature methods*, 15(6):461–468, 2018.

Eric Talevich, A Hunter Shain, Thomas Botton, and Boris C Bastian. CNVkit: genome-wide copy number detection and visualization from targeted DNA sequencing. *PLoS computational biology*, 12(4):e1004873, 2016.
